# New Acyclic Cytotoxic Jasplakinolide Derivative from the Marine Sponge *Jaspis splendens*

**DOI:** 10.3390/md17020100

**Published:** 2019-02-06

**Authors:** Sherif S. Ebada, Werner E. G. Müller, Wenhan Lin, Peter Proksch

**Affiliations:** 1 Institute of Pharmaceutical Biology and Biotechnology, Heinrich-Heine University, Universitaetsstrasse 1, D-40225 Duesseldorf, Germany; 2 Department of Pharmacognosy, Faculty of Pharmacy, Ain-Shams University, Abbasia, Cairo 11566, Egypt; 3 Department of Pharmaceutical Chemistry, Faculty of Pharmacy, Mu’tah University, Al-Karak 61710, Jordan; 4 Institute of Physiological Chemistry, University Medical Center of the Johannes Gutenberg University Mainz, 55128 Mainz, Germany; wmueller@uni-mainz.de; 5 State Key Laboratory of Natural and Biomimetic Drugs, Peking University, Beijing 100083, China; whlin@bjmu.edu.cn

**Keywords:** *Jaspis splendens*, jasplakinolide Z_6_, jasplakinolide Z_5_, cytotoxic activity

## Abstract

A new acylic jasplakinolide congener (**2**), another acyclic derivative requiring revision (**4**), together with two jasplakinolide derivatives including the parent compound jasplakinolide (**1**) were isolated from the Indonesian marine sponge *Jaspis splendens*. The chemical structures of the new and known compounds were unambiguously elucidated based on HRESIMS and exhaustive 1D and 2D NMR spectral analysis as well as a comparison of their NMR data with those of jasplakinolide (**1**). The isolated jasplakinolides inhibited the growth of mouse lymphoma (L5178Y) cells in vitro with IC_50_ values in the low micromolar to nanomolar range.

## 1. Introduction

Jasplakinolide (**1**) (also known as jaspamide) is a cyclodepsipeptide that was first reported in 1986 by Ireland [1] and Crews [2] from the marine sponge *Jaspis* (syn.: *Doryplores*) *splendens* (order Astrophorida, family Ancorinidae). Biosynthetically, jasplakinolide features a polyketide–peptide hybrid derived from the PKS-NRPS pathway [3]. Jasplakinolide revealed potent pharmacological activities such as antifungal [4], anthelmintic [1], insecticidal [2] and most profoundly its highly potent cytotoxic activity with IC_50_ values in the nanomolar range [5]. Since then, an in-depth investigation has been directed toward the identification of its mode of action as a cytotoxic agent, which revealed that **1** exerts its cytotoxic activity by disrupting the actin cytoskeleton as a potent stabilizer of filamentous actin (F-actin) [6]. Subsequently, many research interests have been invested in marine sponges producing cyclodepsipeptides with mixed skeletons structurally related to jasplakinolide, even if they are taxonomically apart from *Jaspis* sponges such as *Geodia* and *Cymbastela* (geodiamolides) [7,8,9,10,11,12] or *Suberites* sponges (seragamides) [13]. In addition to the cyclodepsipeptides obtained from *Jaspis* sponges, some acyclic congeners have been reported that have been considered as plausible hydrolytic products according to the determined biosynthetic gene clusters of chondramide C [14]. These feature three main biosynthetic phases starting with PKS portion formation and ending with the addition of a tyrosine moiety prior to the ring closure creating a macrocycle. To end up with an opened congener, this may be a biosynthetic pathway devoid of the cyclization step or unexpectedly activating a second enzyme cluster to reopen the 19-membered ring at either the ester bond between C-1 and C-15 or the amide bond between C-3 and tyrosine-*N*H [15]. 

In a continuation of our ongoing research targeting new and bioactive cytotoxic jasplakinolide derivatives, we previously explored the extract of three specimens of the Indonesian marine sponge *Jaspis splendens*, affording jasplakinolides Q and R, which were new congeners, together with the parent compound jasplakinolide (**1**) [16]. The remaining semi-pure fractions of the extract were combined and re-fractionated by vacuum liquid chromatography (VLC) using the C18 stationary phase with a gradient elution using water and methanol as a mobile phase. The resulting VLC fractions were then re-evaluated by a de-replication strategy to identify unreported molecular weights of jasplakinolides, which may indicate the existence of new jasplakinolide congeners.

**Figure 1:**
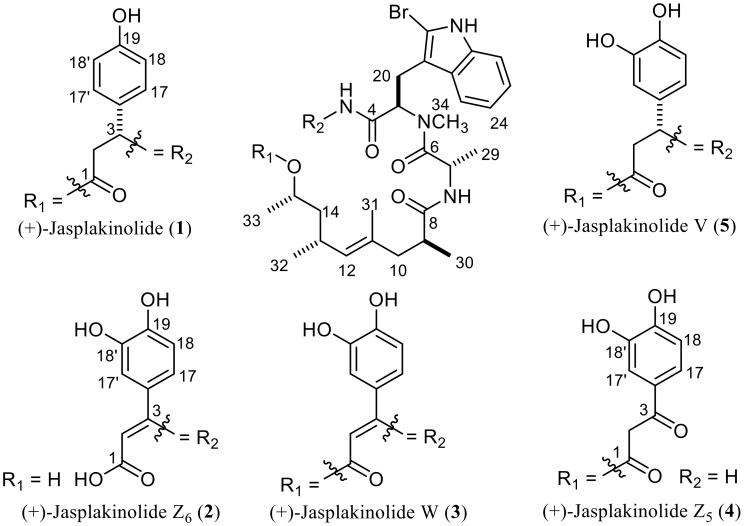
Structures of 1–5.

In this study we report the isolation, identification and bioactivity assessment of a new acyclic natural jasplakinolide (**2**) and a structural revision for a previously reported derivative (**4**) (Figure 1), in addition to two known cyclic derivatives (**1** and **5**).

## 2. Results and Discussion

Compound **2** was obtained as an amorphous solid. The HRESIMS spectrum of **2** revealed pseudomolecular ion peaks at *m/z* 758.2749 [M + NH_4_]^+^ (calcd for C_36_H_49_BrN_5_O_8_; *m/z* 758.2759) and *m/z* 763.2311 [M + Na]^+^ (calcd for C_36_H_45_BrN_4_NaO_8_; *m/z* 763.2313), indicating the molecular formula C_36_H_45_BrN_4_O_8_, which differs from that of **1** by two oxygen atoms [17]. The UV spectrum of **2** revealed an absorption maximum (λ_max_) at 326 nm in addition to those at 220 and 280 nm, suggesting the presence of an acyclic jasplakinolide derivative [17].

**Figure 2:**
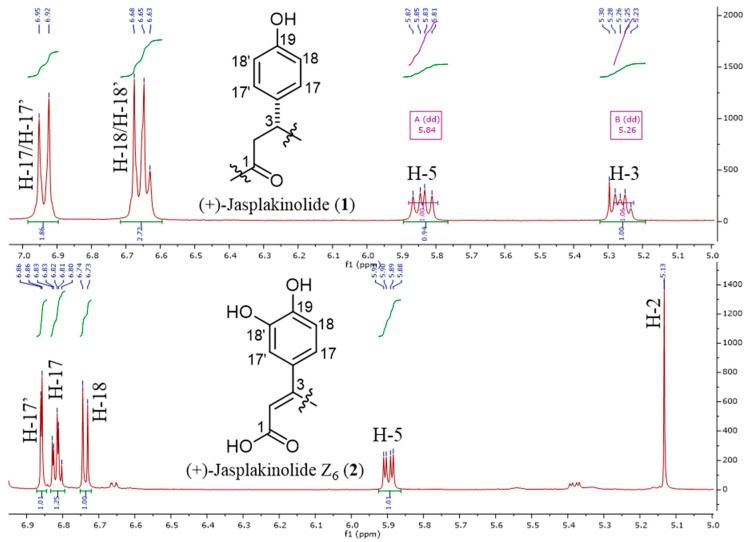
^1^H NMR spectra of **1** and **2** showing differences in aromatic protons at the *β*-tyrosine residue.

The ^1^H and ^13^C NMR data of **2** (Table 1) were similar to those of **1** except for the presence of downfield-shifted aromatic protons of an ABX system, ascribed for a catechol moiety, which replaced those of the AA’BB’ system of **1** (Figure 2). A further difference between the ^1^H NMR spectra of both compounds was the presence of an olefinic singlet at δ_H_ 5.13 in the spectrum of **2,** which was found to be connected to a sp^2^ methine carbon at δ_C_ 99.7, as observed in the HMQC spectrum.

Further spectral information of the structure of **2** was provided by ^1^H NMR measurement in DMSO-*d_6_,* which showed four downfield proton resonances at δ_H_ 11.62 (s), 10.95 (s), 9.41 (br s) and 9.07 (br s). These were assigned to the N*H* signal of the pyrrole ring of tryptophan, a terminal carboxylic acid group of a *β*-tyrosine unit and the two phenolic hydroxyl groups of the catechol moiety, respectively.

Based on previous findings, **2** was primarily suggested to be an acyclic jasplakinolide derivative of (+)-jasplakinolide W (**3**) [17].

**Figure 3:**
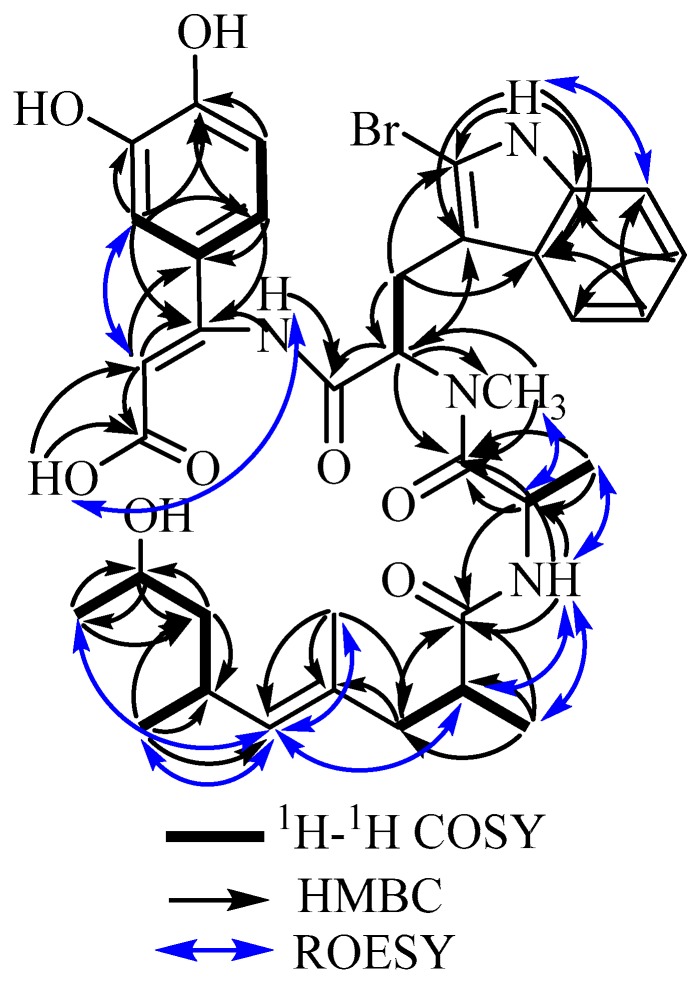
Key ^1^H–^1^H COSY, HMBC and ROESY correlations of **2**.

Further evidence for the structure of **2** was provided via 2D NMR measurements including ^1^H–^1^H COSY, HMBC, HMQC and ROESY experiments (see supplementary materials, Figures S5–S8). Key correlations exhibited in the COSY spectrum confirmed the ABX system of the catechol moiety. The HMBC spectrum showed correlations from the olefinic singlet proton at δ_H_ 5.13 to three quaternary carbons at δ_C_ 168.3, 153.4 and 125.8, which were assigned to C-1, C-3 and C-16, respectively, confirming the presence of a double bond between C-2 and C-3. Further HMBC correlations were found from a broad singlet proton at δ_H_ 6.80 (3–N*H*–4) to two quaternary carbons C-3 and C-4 (δ_C_ 168.3), which indicate the connection between the tryptophan and the *β*-dihydroxyphenyl alanine moiety. Based on the structural evidence from NMR spectra and the molecular formula, **2** was assigned as an acyclic jasplakinolide derivative that features a *β*-dihydroxyphenyl alanine with a double bond between C-2 and C-3 instead of a *β*-tyrosine unit as found in **1**. The stereochemistry of the double bond between C-2 and C-3 was assigned as *cis* according to the priority and based on the strong ROESY correlations (Figure 3, as well as Figure S8a in the supplementary materials) from H-2 to H-17′ and H-17, whereas no correlation was found between H-2 and the amide proton, which connects the *β*-dioxyphenyl alanine group to the brominated tryptophan moiety.

Based on the close similarity of the ROESY spectrum obtained for **2** to those of **1** and **3,** together with the optical rotation (+47.6 (0.6, MeOH)), it was unambiguously confirmed that **2** is an acyclic derivative of **3**, lacking the depside bond between C-1 and C-15. Following the previously reported series of acyclic jasplakinolides [17], **2** was trivially named (+)-jasplakinolide Z_6_.

Compound **4** was obtained as a white amorphous solid and interestingly its HRESIMS spectrum showed pseudomolecular ion peaks at *m/z* 758.2749 [M + NH_4_]^+^ (calcd for C_36_H_49_BrN_5_O_8_; *m/z* 758.2759) and *m/z* 763.2306 [M + Na]^+^ (calcd for C_36_H_45_BrN_4_NaO_8_; *m/z* 763.2302), determining its molecular formula as C_36_H_45_BrN_4_O_8_, identical to that of **2**. The UV spectrum of **4** revealed an additional absorption maximum (λ_max_) at 310.7 nm to the usual maximal absorption wavelengths at 220 and 280 nm found for other derivatives, indicating the presence of an acyclic jasplakinolide congener. Based on the determined molecular formula and UV spectrum of **4** and by comparison to the previously reported jasplakinolides, it was suggested to be the reported derivative (+)-jasplakinolide Z_5_ [17]. 1D and 2D NMR data of **4** revealed a similar pattern of benzenoid moiety in *β*-tyrosine residue with an additional hydroxyl group (catechol ring) with three proton resonances being part of an ABX spin system similar to that found in **2** (Table 1). In addition, the ^1^H NMR data revealed two doublets at δ_H_ 3.87 and δ_H_ 3.73 (d, *J* = 16.5 Hz), which were directly correlated through the HMQC spectrum to a methylene carbon (δ_C_ 45.8). In addition, the gHMBC spectrum of **4** featured clear correlations from a doublet at δ_H_ 3.87 to two carbons at δ_C_ 168.1 and 192.3 with the latter having key correlations to aromatic protons at δ_H_ 7.39 (d, *J* = 2.0 Hz, H-17’), δ_H_ 7.37 (dd, *J* = 8.3, 2.0 Hz, H-17) together with ^4^*J*_H-C_ (*ω*) correlation to the proton signal at δ_H_ 6.82 (d, *J* = 8.3 Hz, H-18), indicating the existence of a ketocarbonyl functionality at C-3, which indicates the cleavage of the 19-membered macrocyclic ring of jasplakinolide at the C3–N bond. By further comparing the 1D and 2D NMR spectral data with those from the literature, **4** was found to be very similar to (+)-jasplakinolide Z_5_. 

**Figure 4:**
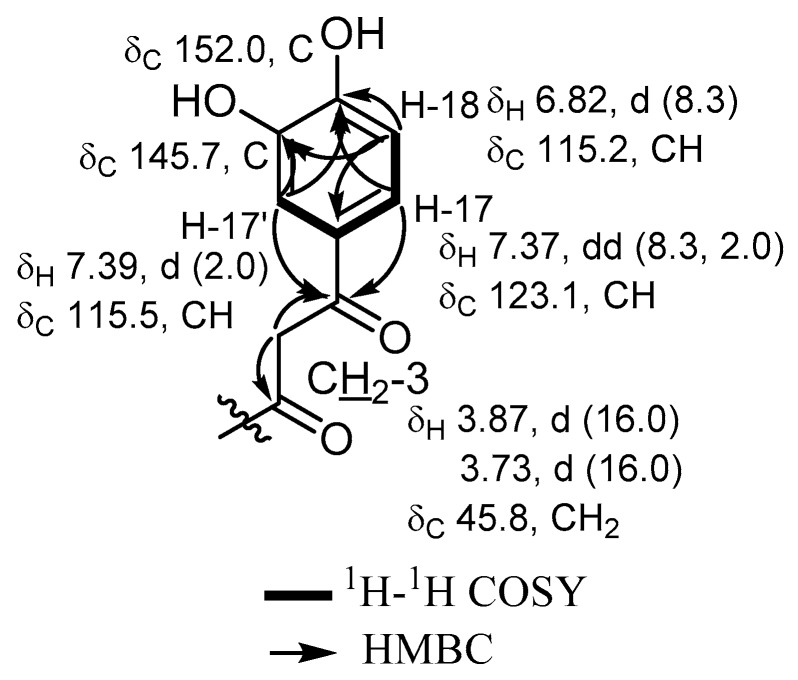
Key ^1^H–^1^H COSY and HMBC correlations of the tyrosine oxygenated moiety of **4**.

However, by a careful inspection of the 2D NMR spectra of **4** and by comparing them to the reported spectral data of (+)-jasplakinolide Z_5_, several discrepancies were clearly observed between the previously reported proton and carbon NMR assignment of the protons and carbons of the aromatic ring of tyrosine (catechol moiety) in the literature [17] and those obtained in this work. The major differences were noticed for the proton and carbon NMR assignments at C-17’, C-18 and C-19 (Figure 4). Further confirmation of the assignment presented in this work was provided by 2D NMR data including ^1^H–^1^H COSY and gHMBC correlations, which revealed clear ^3^*J*_H-C_ correlations from H-17 and H-17’ to an oxygenated aromatic carbon at δ_C_ 152.0 (C-19), in addition to the correlations from H-18 and H-17’ to another oxygen-bearing aromatic carbon at δ_C_ 145.7 (C-18’). All this evidence supported the final assignment of the revised NMR data of (+)-jasplakinolide Z_5_ [17] as depicted in Table 1 and Figure 4.

Based on the plausible common biosynthetic pathway of all isolated compounds and the parent compound jasplakinolide (**1**), they are expected to have the same absolute configuration.

All the isolated compounds were assessed for their antiproliferative activity against mouse lymphoma (L5178Y) cells using the microculture tetrazolium (MTT assay) and compared to kahalalide F as a positive control (IC_50_ = 4.3 µM). Results revealed that all the isolated compounds possess potent cytotoxic activity with IC_50_ values in the low micromolar to nanomolar range. Among the tested compounds, **2** was the least potent (IC_50_ = 3.2 µM) derivative compared to **4**, **5** and **1,** which showed IC_50_ values in the nanomolar range (<100 nM). This indicates that the 19-membered ring of jasplaklinolide is not a major structural requirement for its activity. The main factor that might influence the antiproliferative activity of the acyclic jasplakinolide congeners is its lipophilic character, as noticed when comparing **2** with **4**, where the former has a free carboxylic acid functionality imparting higher polarity to the molecule compared to **4**. Further proof was provided by a comparison of the antiproliferative activity of three acyclic jasplakinolides Z_1_–Z_3_ against a panel of human cancer cells, where the latter two derivatives are the methyl and ethyl esters of the former one, respectively [17]. Results also revealed that the hydrolysis of the depside bond, yielding a free carboxylic acid group significantly diminished its activity, which was almost turned back to be equivalent to the parent compound by converting the carboxylic acid group into an ester functionality. This can be attributed to the increased lipophilicity of the ester derivatives that may facilitate their cellular uptake and hence recuperate the activity of the compound.

Adding to the main structure-activity relationship (SAR) characteristics influencing the antiproliferative activity of jasplakinolide [17], the 19-membered macrocyclic ring is not necessary for activity, since it can be opened to yield acyclic congeners with equivalent activities, provided that they keep the lipophilic character sufficient to facilitate cellular uptake.

## 3. Experimental Section 

### 3.1. General Experimental Procedures 

Vacuum liquid chromatography (VLC) was carried out using C_18_ reversed-phase and a gradient elution was applied, going from water to methanol with a 10% gradient interval. A Perkin-Elmer-241 MC polarimeter (Perkin-Elmer, Akron, Ohio, USA) was used for measuring the optical rotation. LRESIMS was recorded on an LCMS HP1100 Agilent Finnigan LCQ Deca XP Thermoquest machine (Thermo Electron Corporation, San Jose, California, USA) and HRESIMS were determined on a FTHRMS-Orbitrap (Thermo Fischer Scientific, Bremen, Germany) mass spectrometer. For analytical HPLC analysis, samples were injected into a Dionex Ultimate 3000 LC system equipped with a photodiode array detector (UVD340S) (Dionex, Munich, Germany) with detection channels at 235, 254, 280 and 340 nm. Ready-made separation columns (125 L × 4 mm ID) were prefilled with Eurosphere-10C_18_ (Knauer, Berlin, Germany) and used a linear gradient from 90% H_2_O (pH 2.0) to 100% MeOH over 40 min with a flow rate of 1 mL/min. TLC analysis was carried out using aluminum sheets precoated with silica gel 60 F_254_ (Merck, Darmstadt, Germany). 

Preparative HPLC separations were performed on a LaChrom-Merck Hitachi HPLC system (Tokyo, Japan), pump L-7100(Hitachi, Tokyo, Japan), UV detector L-7400 (Hitachi, Tokyo, Japan) using a column (Knauer, 300 L × 8 mm ID, prefilled with 100 C_18_ Eurosphere, flow rate 5 mL/min, UV detection at 280 nm), and the solvent system consisted of a linear gradient of MeOH and nanopure H_2_O running 40%–90% MeOH over 30 min.

1D (^1^H and ^13^C NMR) and 2D NMR (COSY, gHMBC, gHMQC and ROESY) spectra were recorded on Bruker AVANCE DMX 600 or 300 spectrometers (Bruker, Fällanden, Switzerland) using chloroform-*d*, DMSO-*d_6_* or methanol-*d_4_* as solvents (Sigma-Aldrich, Taufkirchen, Germany).

### 3.2. Biological Material 

In August 2008, sponge specimens were collected on three neighboring islands of East Kalimantan (Indonesia), namely Samama, Panjang and Shoal Islands, at 10-meter depths. Numbers of voucher specimens are RMNH Por. 4234, 4266 and 4299, respectively. They were taxonomically identified as *Jaspis splendens* (order Astrophorida, family Ancorinidae) by Dr. Nicole de Voogd at the National Museum of Natural History, Leiden, Netherlands, where the voucher specimens are kept. HPLC and LCMS analyses of the three samples revealed that they were identical with regard to their peptide derivatives. Hence, the materials were combined in order to obtain a sufficient amount of compounds for subsequent structure elucidation.

### 3.3. Extraction and Isolation 

The animal was freeze-dried and the material (500 g) was extracted with methanol (3 × 2 L) and filtered. The extracts were then combined, evaporated under reduced pressure to dryness and processed as follows. The methanolic extract (80 g) was dissolved in water and partitioned with *n*-hexane, ethyl acetate and then *n*-butanol. The bioactive ethyl acetate soluble fraction (2 g) was loaded on C18 reversed-stationary phase and subjected to VLC using a linear gradient from water to MeOH to afford 11 fractions (Fr. I-XI). Fr. VIII, eluted with methanol:water (7:3) (670 mg), revealed several peaks with UV absorption spectra similar to that of jasplakinolide. An aliquot of 300 mg of Fr. VIII was further purified by preparative HPLC, equipped with a C_18_ ready-made column using a gradient elution of MeOH and nanopure H_2_O, 40%–90% MeOH over 30 min, to give **1** (140.0 mg), **2** (46.8 mg), **4** (33.7 mg) and **5** (21.0 mg).

*(+)-Jasplakinolide Z_6_* (**2**): amorphous yellow solid; ${\left[\alpha \right]}_{D}^{20}$ **+**47.6° (*c* 0.60, MeOH); UV (MeOH) λ_max_ 220, 280, 326.1 nm; ^1^H-NMR and ^13^-C NMR see Table 1; HRESIMS *m/z* 758.2749 [M + NH_4_]^+^ (calcd for C_36_H_49_BrN_5_O_8_; *m/z* 758.2759) and *m/z* 763.2311 [M + Na]^+^ (calcd for C_36_H_45_BrN_4_NaO_8_; *m/z* 763.2313).

*(+)-Jasplakinolide Z_5_* (**4**): white amorphous solid; ${\left[\alpha \right]}_{D}^{20}$ **+**58.0° (*c* 0.40, MeOH); UV (MeOH) λ_max_ 220, 280, 310.7 nm; ^1^H-NMR and ^13^-C NMR see Table 1; HRESIMS *m/z* 758.2749 [M + NH_4_]^+^ (calcd for C_36_H_49_BrN_5_O_8_; *m/z* 758.2759) and *m/z* 763.2306 [M + Na]^+^ (calcd for C_36_H_45_BrN_4_NaO_8_; *m/z* 763.2302).

### 3.4. Cell Proliferation Assay

Cytotoxicity was tested against L5178Y mouse lymphoma cells using the microculture tetrazolium (MTT) assay as described earlier [18,19]. All experiments were carried out in triplicate and repeated three times. As controls, media with 0.1% EGMME/DMSO was included in the experiments.

## Figures and Tables

**Table 1:** ^1^H and ^13^C NMR data of **2** and **4**.

pos.	2		4	
	δ_H_ (*J* in Hz) *^a^*	δ_C_, type *^a,c,d^*	δ_H_ (*J* in Hz) *^b^*	δ_C_, type *^b,c,d^*
1		168.3, CO		168.5, CO
2	5.13, s	99.7, CH	3.87, d (16.0)	45.8, CH_2_
			3.73, d (16.0)	
3		153.4, C		192.7, CO
4		168.3, C		173.9, CO
5	5.90, dd (11.5, 4.5)	56.1, CH	5.67, dd (11.4, 4.8)	56.6, CH
6		173.3, CO		174.7, CO
7	4.65, p (6.7)	44.9, CH	4.40, q (6.9)	45.9, CH
8		174.1, CO		178.2, CO
9	2.64, ddd (11.5, 6.9, 2.1)	36.3, CH	2.39, dq (14.1, 6.9)	39.0, CH
10	2.22, dd (16.8. 11.6)	40.0, CH_2_	2.20, dd (13.5, 6.7)	44.0, CH_2_
	1.69, d (16.8)		1.87, dd (13.5, 8.1)	
11		133.0, C		132.2, C
12	4.73, m	125.8, CH	4.87, d (9.7)	133.3, CH
13	2.30, dq (10.8, 5.4, 4.5)	28.8, CH	2.31, dq (14.0, 6.9)	29.3, CH
14	1.46, m	43.2, CH_2_	1.52, dd (14.0, 7.3)	43.7, CH_2_
	1.29, ddd (12.8, 9.9, 3.9)		1.33, dd (14.0, 6.7)	
15	4.71, m	68.8, CH	4.90, m	71.3, CH
16		125.8, C		128.8, C
17	6.82, dd (8.2, 2.2)	119.0, CH	7.37, dd (8.3, 2.0)	123.1, CH
17’	6.86, d (2.2)	115.1, CH	7.39, d (2.0)	115.5, CH
18	6.74, d (8.2)	115.0, CH	6.82, d (8.3)	115.2, CH
18’		144.6, C		145.7, C
19		147.6, C		152.0, C
20	3.16, dd (15.2, 4.4)	22.1, CH_2_	3.39, dd (15.3, 4.9)	24.0, CH_2_
	3.03, dd (15.1, 11.6)		3.15, dd (15.3, 11.5)	
21		109.4, C		109.5, C
22		127.2, C		127.8, C
23	7.58, d (8.0)	118.0, CH	7.48, d (8.0)	118.3, CH
24	6.99, td (7.2, 0.9)	119.9, CH	7.00, t (8.0)	119.7, CH
25	7.06, td (7.2, 0.9)	121.5, CH	7.05, t (8.0)	122.1, CH
26	7.24, d (8.0)	110.6, CH	7.23, d (8.0)	111.0, CH
27		136.1, C		137.0, C
28		108.9, C		111.0, C
29	0.42, d (6.7)	17.2, CH_3_	0.58, d (6.9)	15.7, CH_3_
30	0.91, d (6.9)	20.5, CH_3_	0.96, d (6.9)	16.8, CH_3_
31	1.50, s	18.3, CH_3_	1.48, s	15.6, CH_3_
32	0.82, d (6.7)	22.2, CH_3_	0.82, d (6.6)	20.6, CH_3_
33	1.11, d (6.3)	18.8, CH_3_	1.16, d (6.2)	19.9, CH_3_
34	2.91, s	31.0, CH_3_	3.02, s	31.7, CH_3_
N*H*–pyrrole	11.62, br s			
7–N*H*–8	7.61, d (7.3)			
3–N*H*–4	6.80, br s			
–COOH	10.95, br s			
OH–18’	9.07, br s			
OH–19	9.41, br s			

*^a^*Measured in DMSO-*d_6_* at 600 and 150 MHz. *^b^* Measured in CDCl_3_ at 600 and 150 MHz. *^c^* Carbon type is determined from gHMQC data. *^d^*Shifts are assigned on the basis of gHMQC and gHMBC data.

## Supplementary Materials

The following are available online at http://www.mdpi.com/1660-3397/17/2/100/s1, Figure S1: HPLC chromatogram of **2**; Figure S2: HRESIMS spectrum of **2**; Figure S3: ^1^H-NMR spectrum of **2** in DMSO-*d_6_*; Figure S3a: ^1^H-NMR spectrum of **2** in DMSO-*d_6_*; Figure S4: ^13^C-NMR spectrum of **2** in MeOH-*d_4_*; Figure S4a: ^13^C-NMR spectrum of **2** in MeOH-*d_4_*; Figure S5: ^1^H-^1^H COSY spectrum of **2** in DMSO-*d_6_*; Figure S5a: ^1^H-^1^H COSY spectrum of **2** in DMSO-*d_6_*; Figure S6: gHMBC spectrum of **2** in DMSO-*d_6_*; Figure S6a: gHMBC spectrum of **2** in DMSO-*d_6_*; Figure S7: gHMQC spectrum of **2** in DMSO-*d_6_*; Figure S7a: gHMQC spectrum of **2** in DMSO-*d_6_*; Figure S8: ROESY spectrum of **2** in DMSO-*d_6_*; Figure S8a: ROESY spectrum of **2** in DMSO-*d_6_*; Figure S9: HPLC chromatogram of **4**; Figure S10: HRESIMS spectrum of **4**; Figure S11: ^1^H-NMR spectrum of **4** in CDCl_3_; Figure S11a: ^1^H-NMR spectrum of **4** in CDCl_3_; Figure S12: ^13^C-NMR spectrum of **4** in CDCl_3_; Figure S13: ^1^H-^1^H COSY spectrum of **4** in CDCl_3_; Figure S14: gHMBC spectrum of **4** in CDCl_3_; Figure S15: gHMQC spectrum of **4** in CDCl_3_.
