# Supplementary material for: New Acyclic Cytotoxic Jasplakinolide Derivative from the Marine Sponge Jaspis splendens
Source: Mar Drugs. 2019 Feb 6;17(2):100. doi: 10.3390/md17020100 (PMC6409940; doi:10.3390/md17020100)
Supplement: Supplementary file 1 [file marinedrugs-17-00100-s001.pdf]

Supplementary Materials for

New Acyclic Cytotoxic Jasplakinolide Derivative from the Marine Sponge *Jaspis splendens*

Sherif S. Ebada[1,2,3,*], Werner E. G. Müller[4], Wenhan Lin[5], Peter Proksch[1,*]

[1] Institute of Pharmaceutical Biology and Biotechnology, Heinrich-Heine University, Universitaets- strasse 1, D-40225 Duesseldorf, Germany; E-mail: proksch@hhu.de

[2] Department of Pharmacognosy, Faculty of Pharmacy, Ain-Shams University, Abbasia, 11566 Cairo, Egypt; E-mail: sherif_elsayed@pharma.asu.edu.eg

[3] Department of Pharmaceutical Chemistry, Faculty of Pharmacy, Mu'tah University, 61710 Al-Karak, Jordan; E-mail: ss_ebada@mutah.edu.jo

[4] Institute of Physiological Chemistry, University Medical Center of the Johannes Gutenberg University Mainz, 55128 Mainz, Germany; E-mail: wmueller@uni-mainz.de

[5] State Key Laboratory of Natural and Biomimetic Drugs, Peking University, Beijing 100083, China; E-mail: whlin@bjmu.edu.cn

* Authors to whom correspondence should be addressed; E-mail: sherif_elsayed@pharma.asu.edu.eg; ss_ebada@mutah.edu.jo (S.S.E.); proksch@uni-duesseldorf.de (P.P.); Tel.: +49211-81-14163. Fax: +49211-81-11923

ABSTRACT

A new acylic jasplakinolide congener (**2**), another acyclic derivative requiring revision (**4**), together with two jasplakinolide derivatives including the parent compound jasplakinolide (**1**) were isolated from the Indonesian marine sponge *Jaspis splendens*. The chemical structures of the new and known compounds were unambiguously elucidated based on HRESIMS and exhaustive 1D and 2D NMR spectral analysis as well as comparison of their NMR data with those of jasplakinolide (**1**). The isolated jasplakinolides inhibited the growth of the mouse lymphoma (L5178Y) cells *in vitro* with IC_{50} values in the low micromolar to nanomolar range.

Keywords: *Jaspis splendens*, jasplakinolide Z_6, jasplakinolide Z_{5a}, cytotoxic activity.

Contents of Supplementary Materials

#	Contents	Page
1	Figure S1. HPLC chromatogram of **2**	S3
2	Figure S2. HRESIMS spectrum of **2**	S3
3	Figure S3. ^{1}H-NMR spectrum of **2** in DMSO-d_6	S4
4	Figure S3a. ^{1}H-NMR spectrum of **2** in DMSO-d_6	S5
5	Figure S4. ^{13}C-NMR spectrum of **2** in MeOH-d_4	S6
6	Figure S4a. ^{13}C-NMR spectrum of **2** in MeOH-d_4	S7
7	Figure S5. ^{1}H–^{1}H COSY spectrum of **2** in DMSO-d_6	S8
8	Figure S5a. ^{1}H–^{1}H COSY spectrum of **2** in DMSO-d_6	S9
9	Figure S6. gHMBC spectrum of **2** in DMSO-d_6	S10
10	Figure S6a. gHMBC spectrum of **2** in DMSO-d_6	S11
11	Figure S7. gHMQC spectrum of **2** in DMSO-d_6	S12
12	Figure S7a. gHMQC spectrum of **2** in DMSO-d_6	S13
13	Figure S8. ROESY spectrum of **2** in DMSO-d_6	S14
14	Figure S8a. ROESY spectrum of **2** in DMSO-d_6	S15
15	Figure S9. HPLC chromatogram of **4**	S16
16	Figure S10. HRESIMS spectrum of **4**	S16
17	Figure S11. ^{1}H-NMR spectrum of **4** in $CDCl_3$	S17
18	Figure S11a. ^{1}H-NMR spectrum of **4** in $CDCl_3$	S18
19	Figure S12. ^{13}C-NMR spectrum of **4** in $CDCl_3$	S19
20	Figure S13. ^{1}H–^{1}H COSY spectrum of **4** in $CDCl_3$	S20
21	Figure S14. gHMBC spectrum of **4** in $CDCl_3$	S21
22	Figure S15. gHMQC spectrum of **4** in $CDCl_3$	S22

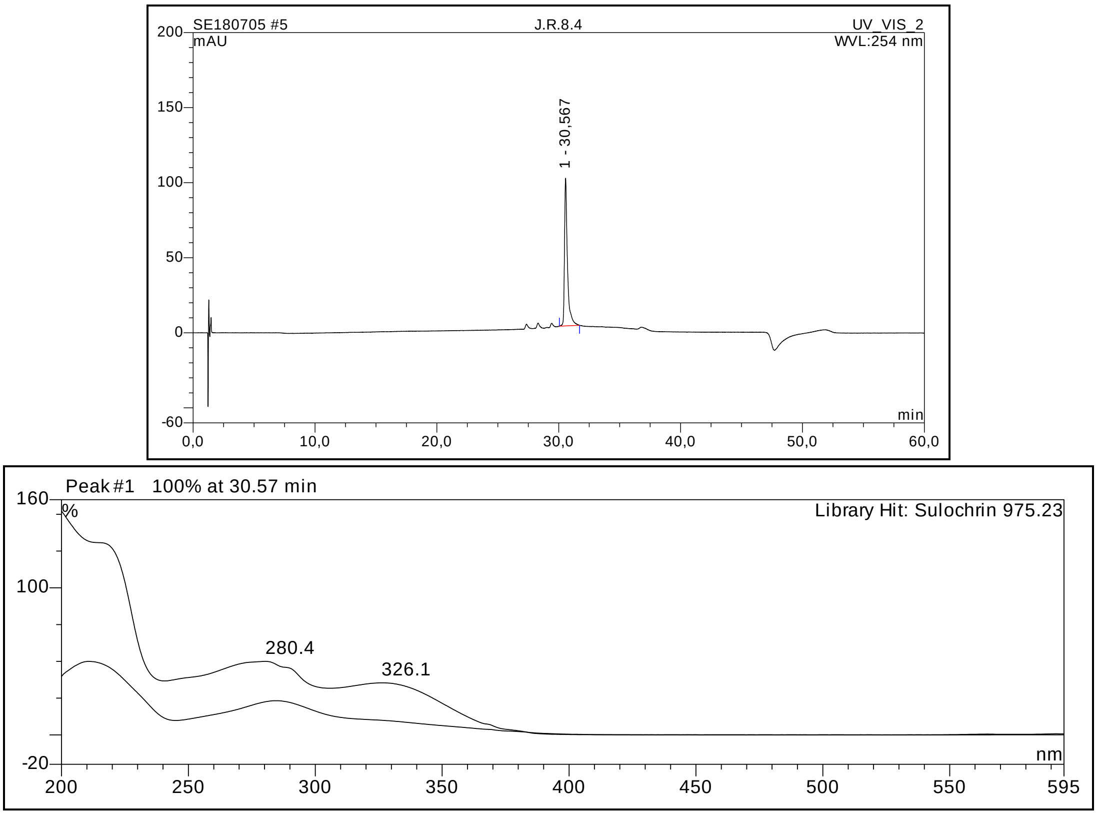

Figure S1. HPLC chromatogram of **2**

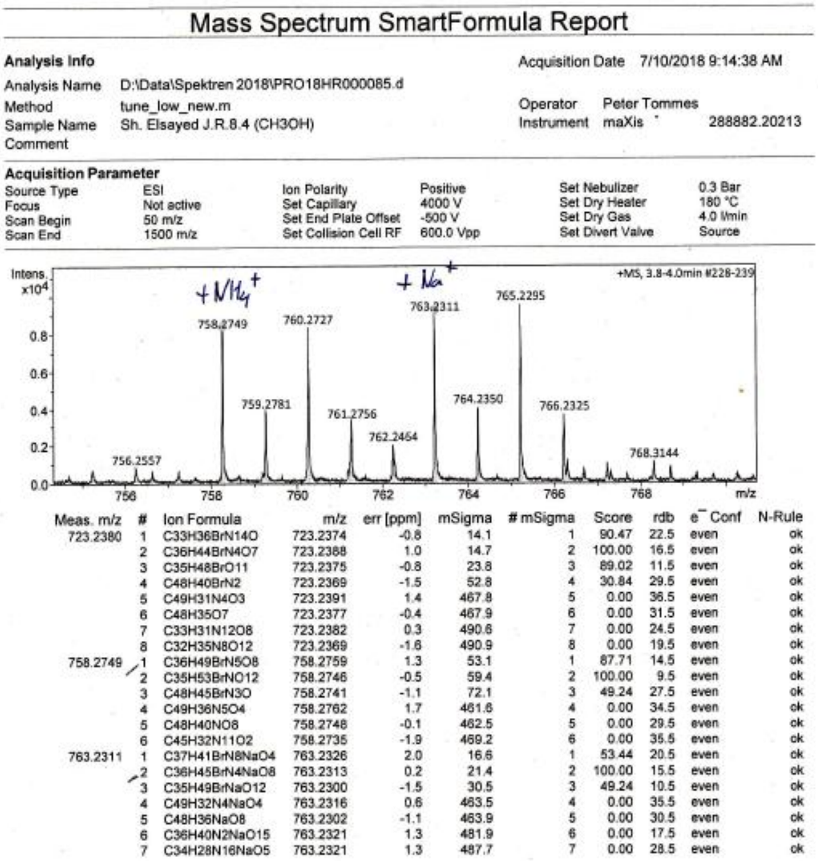

Mass Spectrum SmartFormula Report

Analysis Info

Analysis Name	D:\Data\Spektren 2018\PRO18HR000085.d	Acquisition Date	7/10/2018 9:14:38 AM
Method	tune_low_new.m	Operator	Peter Tommes
Sample Name	Sh. Elsayed J.R.8.4 (CH3OH)	Instrument	maXis 288882.20213
Comment			

Acquisition Parameter

Source Type	ESI	Ion Polarity	Positive	Set Nebulizer	0.3 Bar
Focus	Not active	Set Capillary	4000 V	Set Dry Heater	180 °C
Scan Begin	50 m/z	Set End Plate Offset	-500 V	Set Dry Gas	4.0 l/min
Scan End	1500 m/z	Set Collision Cell RF	600.0 Vpp	Set Divert Valve	Source

Meas. m/z	#	Ion Formula	m/z	err [ppm]	mSigma	# mSigma	Score	rdb	e⁻ Conf	N-Rule
723.2380	1	C33H36BrN14O	723.2374	-0.8	14.1	1	90.47	22.5	even	ok
	2	C36H44BrN4O7	723.2388	1.0	14.7	2	100.00	16.5	even	ok
	3	C35H48BrO11	723.2375	-0.8	23.8	3	89.02	11.5	even	ok
	4	C48H40BrN2	723.2369	-1.5	52.8	4	30.84	29.5	even	ok
	5	C49H31N4O3	723.2391	1.4	467.8	5	0.00	36.5	even	ok
	6	C48H35O7	723.2377	-0.4	467.9	6	0.00	31.5	even	ok
	7	C33H31N12O8	723.2382	0.3	490.6	7	0.00	24.5	even	ok
	8	C32H35N8O12	723.2369	-1.6	490.9	8	0.00	19.5	even	ok
758.2749	1	C36H49BrN5O8	758.2759	1.3	53.1	1	87.71	14.5	even	ok
	2	C35H53BrNO12	758.2746	-0.5	59.4	2	100.00	9.5	even	ok
	3	C48H45BrN3O	758.2741	-1.1	72.1	3	49.24	27.5	even	ok
	4	C49H36N5O4	758.2762	1.7	461.6	4	0.00	34.5	even	ok
	5	C48H40NO8	758.2748	-0.1	462.5	5	0.00	29.5	even	ok
	6	C45H32N11O2	758.2735	-1.9	469.2	6	0.00	35.5	even	ok
763.2311	1	C37H41BrN8NaO4	763.2326	2.0	16.6	1	53.44	20.5	even	ok
	2	C36H45BrN4NaO8	763.2313	0.2	21.4	2	100.00	15.5	even	ok
	3	C35H49BrNaO12	763.2300	-1.5	30.5	3	49.24	10.5	even	ok
	4	C49H32N4NaO4	763.2316	0.6	463.5	4	0.00	35.5	even	ok
	5	C48H36NaO8	763.2302	-1.1	463.9	5	0.00	30.5	even	ok
	6	C36H40N2NaO15	763.2321	1.3	481.9	6	0.00	17.5	even	ok
	7	C34H28N16NaO5	763.2321	1.3	487.7	7	0.00	28.5	even	ok

Figure S2. HRESIMS spectrum of **2**

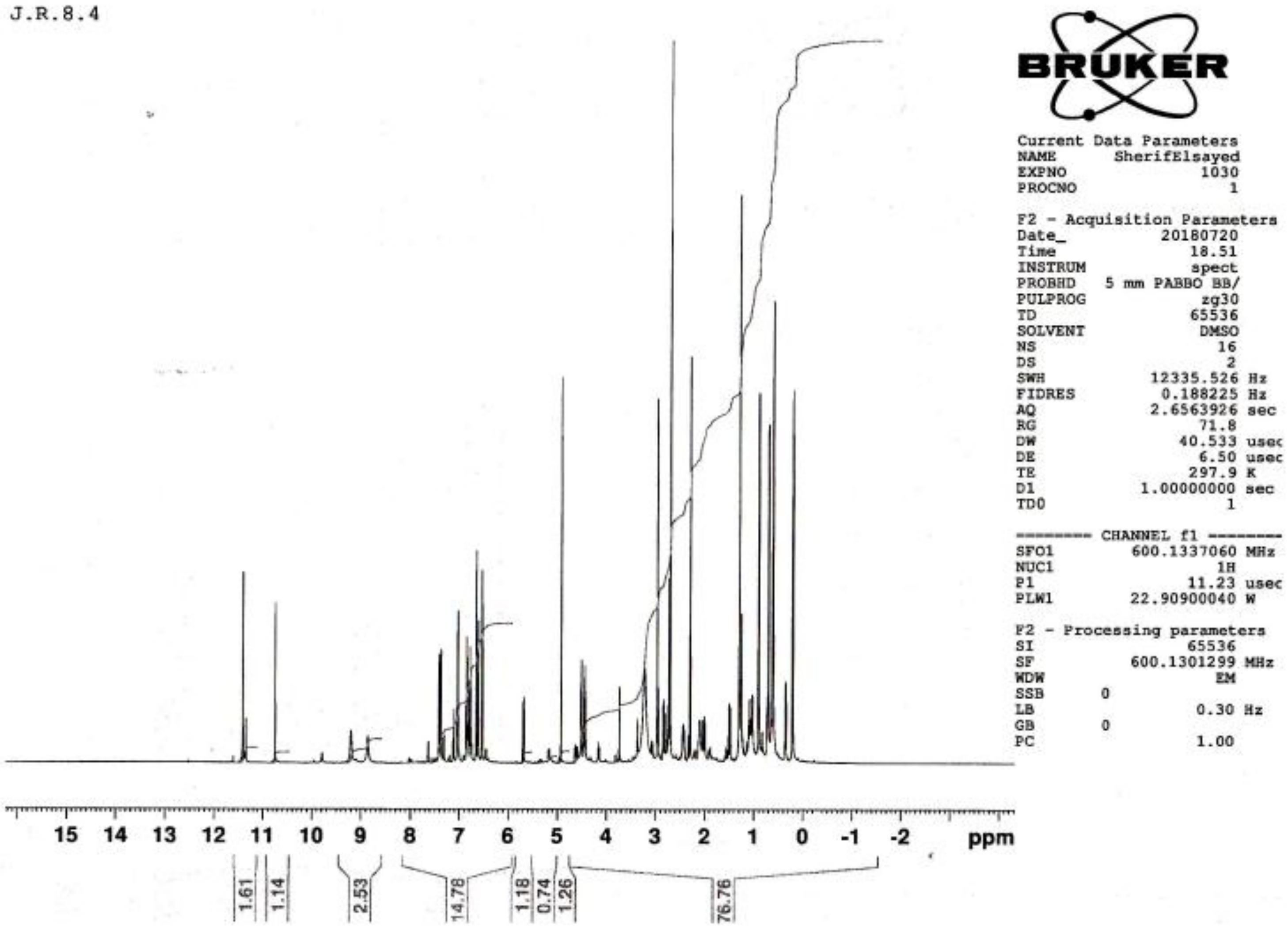

Figure S3. ^{1}H-NMR spectrum of **2** in DMSO-d_6

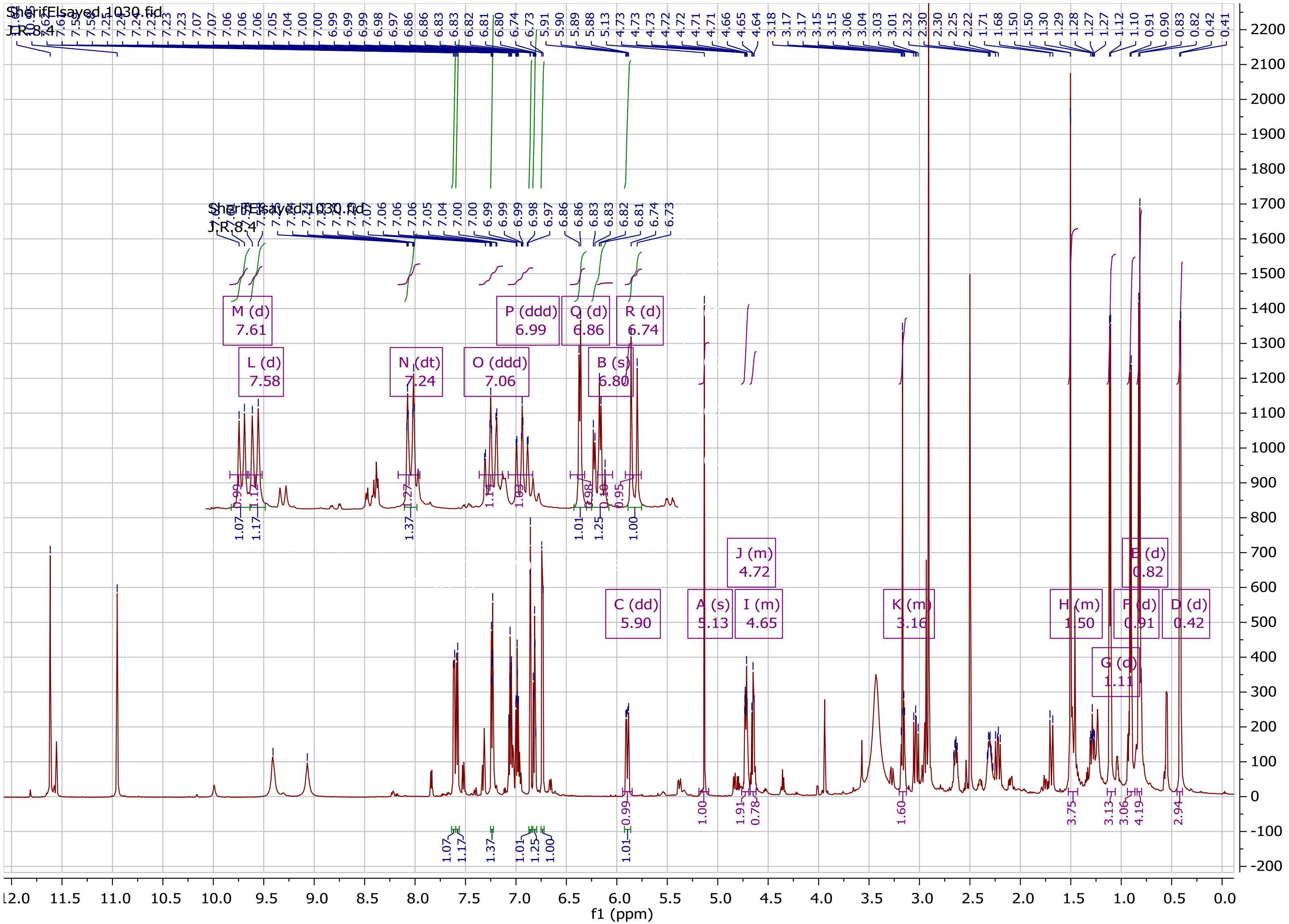

Figure S3a. ^{1}H-NMR spectrum of **2** in DMSO-d_6

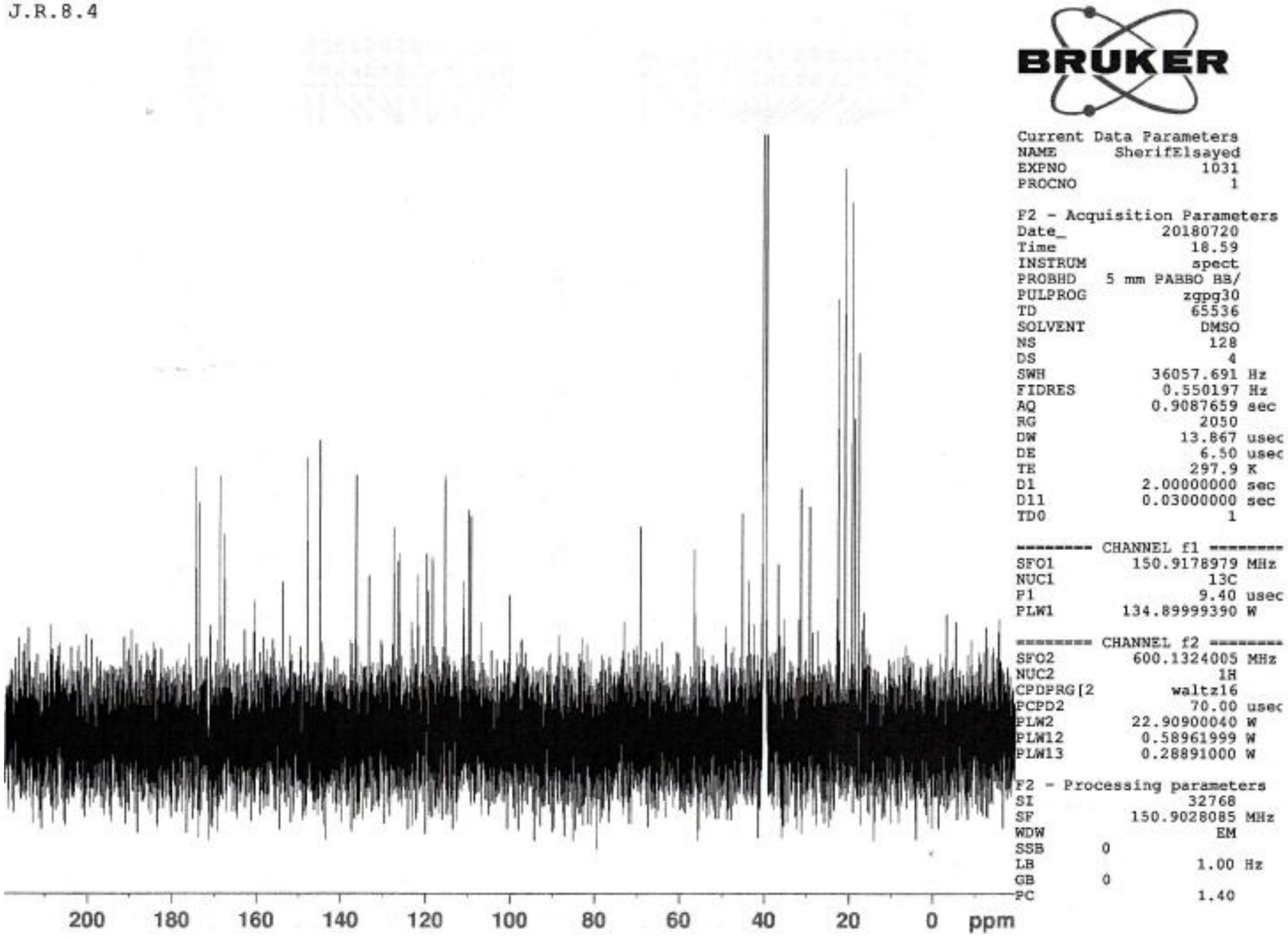

Figure S4. ^{13}C-NMR spectrum of **2** in DMSO-d_6

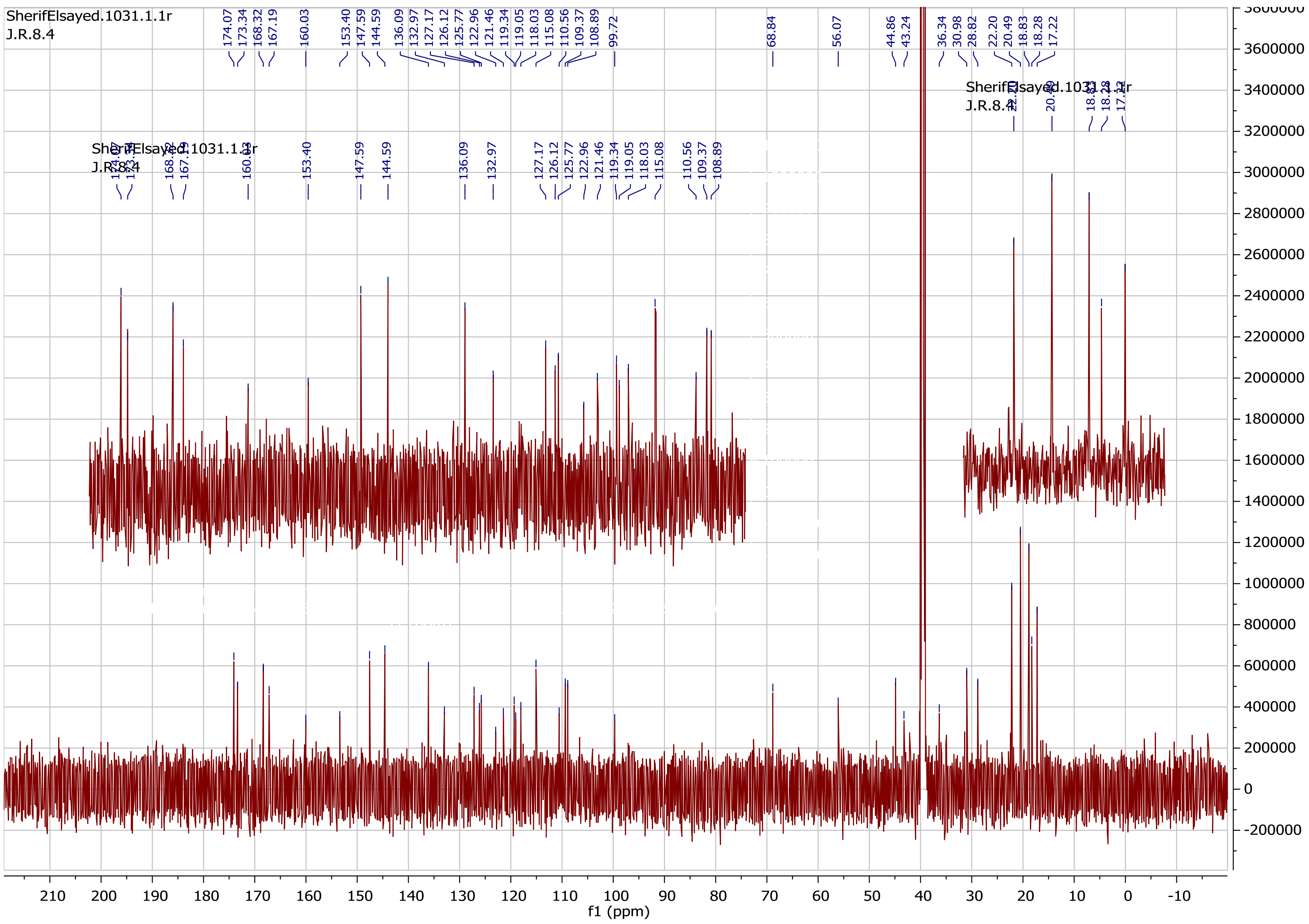

Figure S4a. ^{13}C-NMR spectrum of **2** in DMSO-d_6

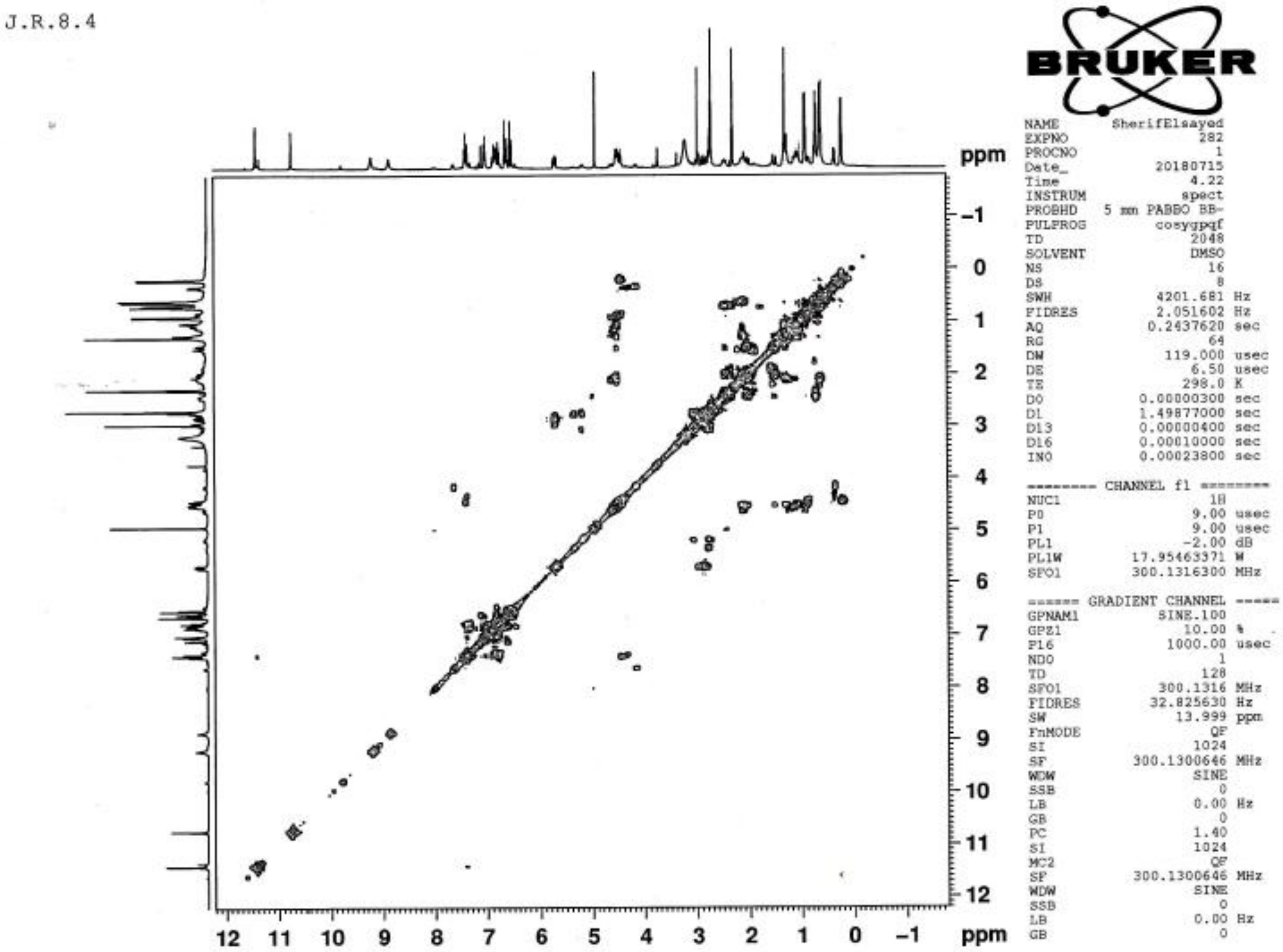

Figure S5. ^{1}H–^{1}H COSY spectrum of **2** in DMSO-d_6

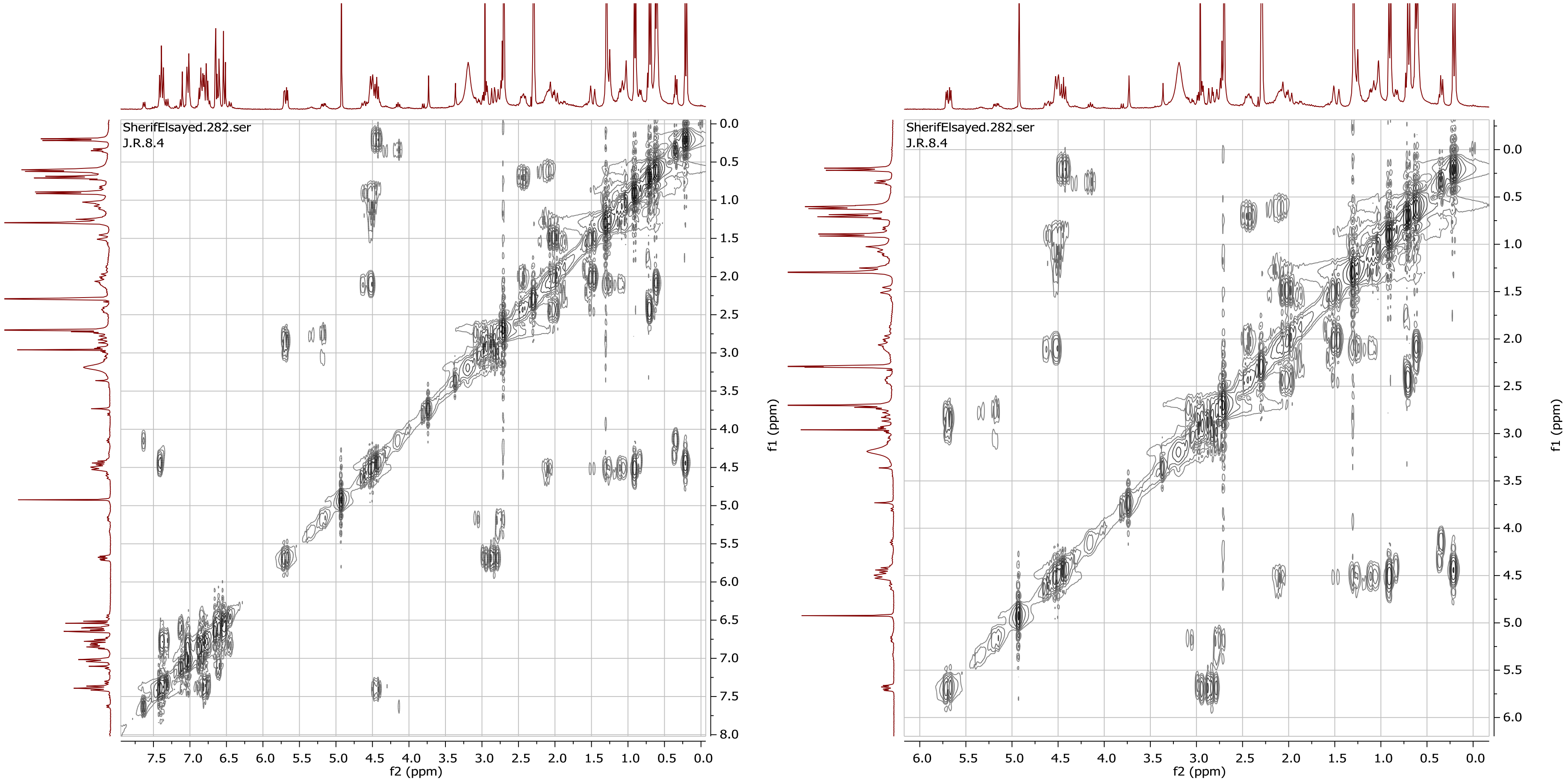

Figure S5a. ^{1}H–^{1}H COSY spectrum of **2** in DMSO-d_6

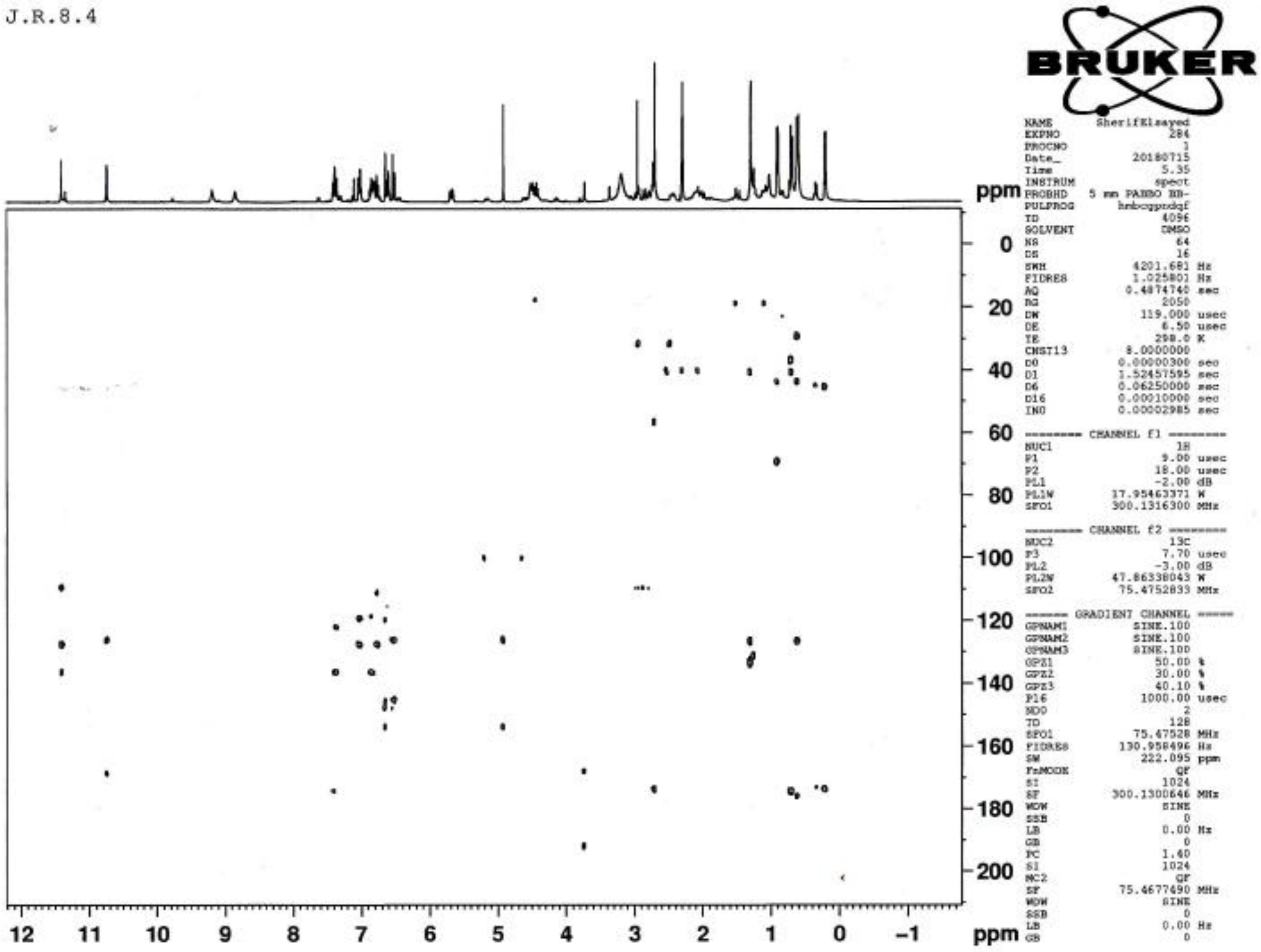

Figure S6. gHMBC spectrum of **2** in DMSO-d_6

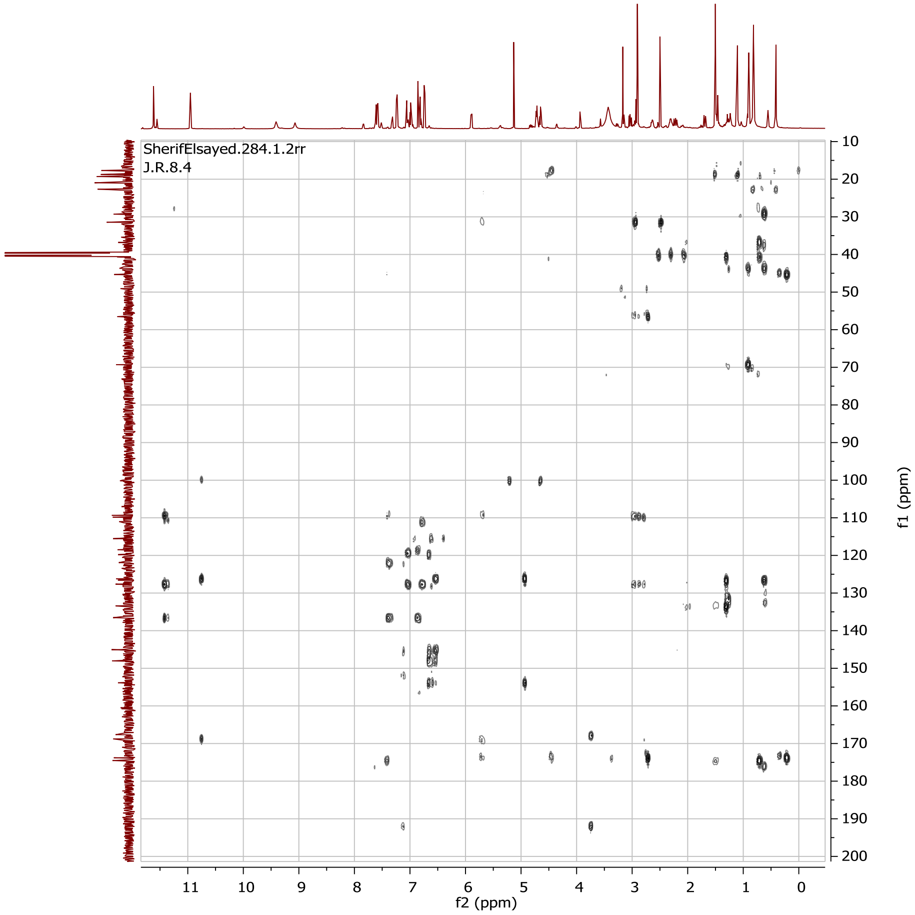

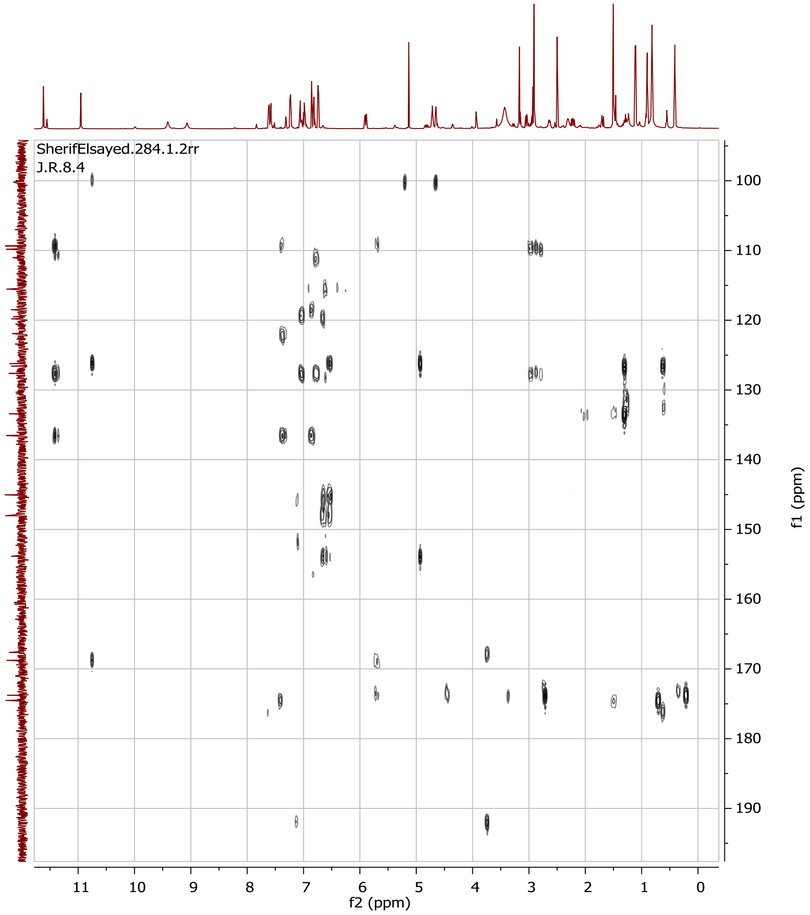

Figure S6a. gHMBC spectrum of **2** in DMSO-d_6

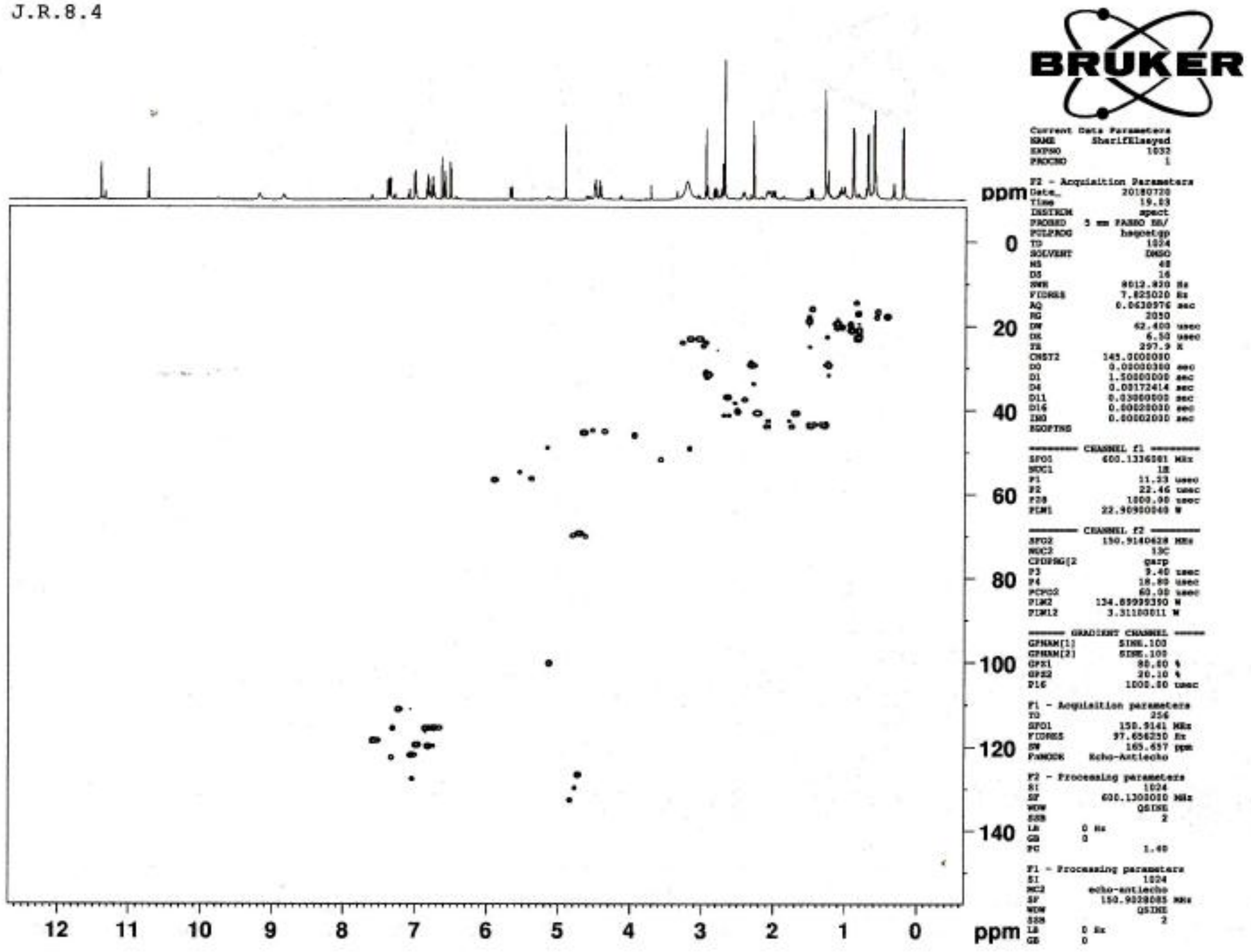

Figure S7. gHMQC spectrum of **2** in DMSO-d_6

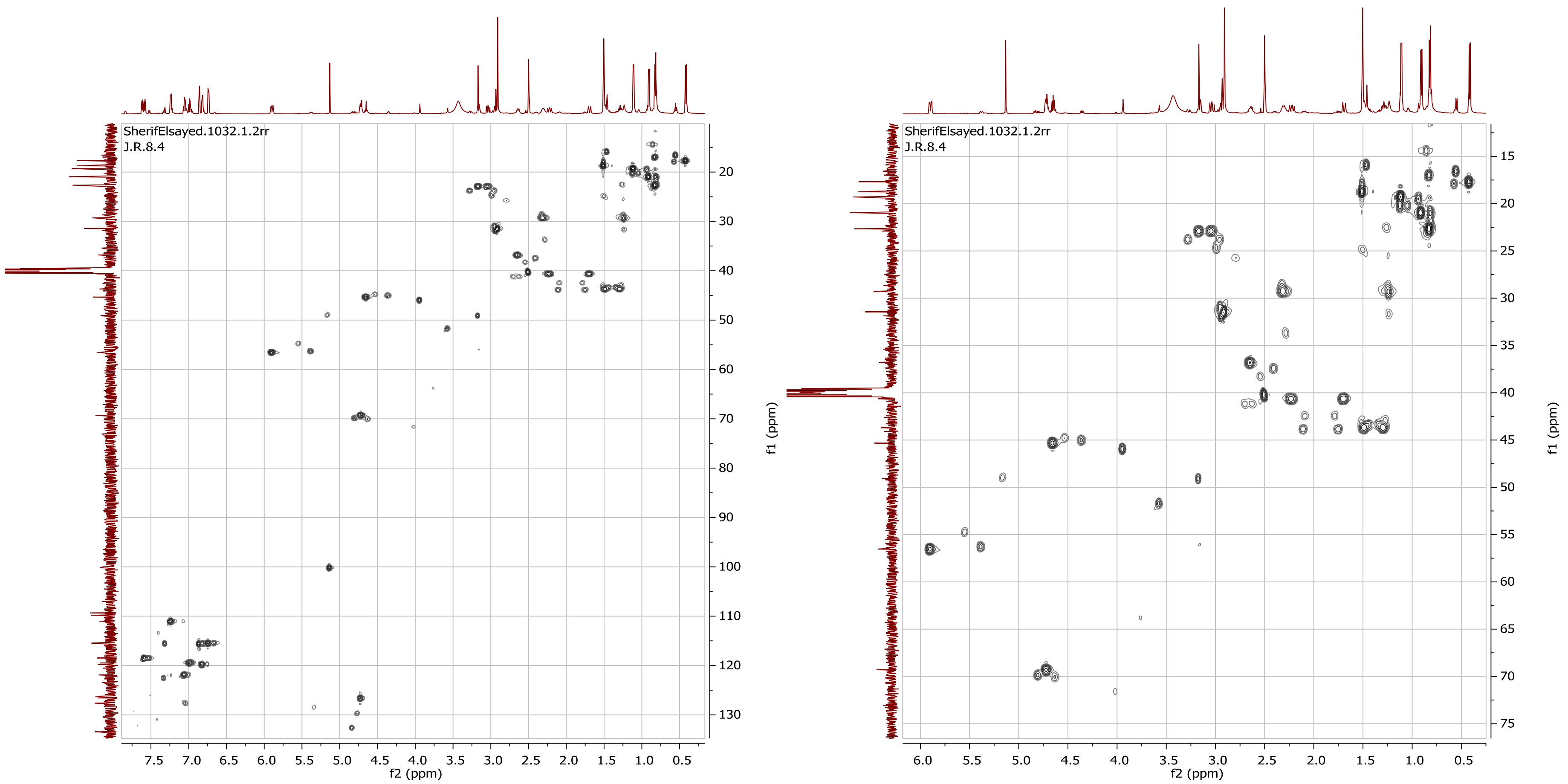

Figure S7a. gHMQC spectrum of **2** in DMSO-d_6

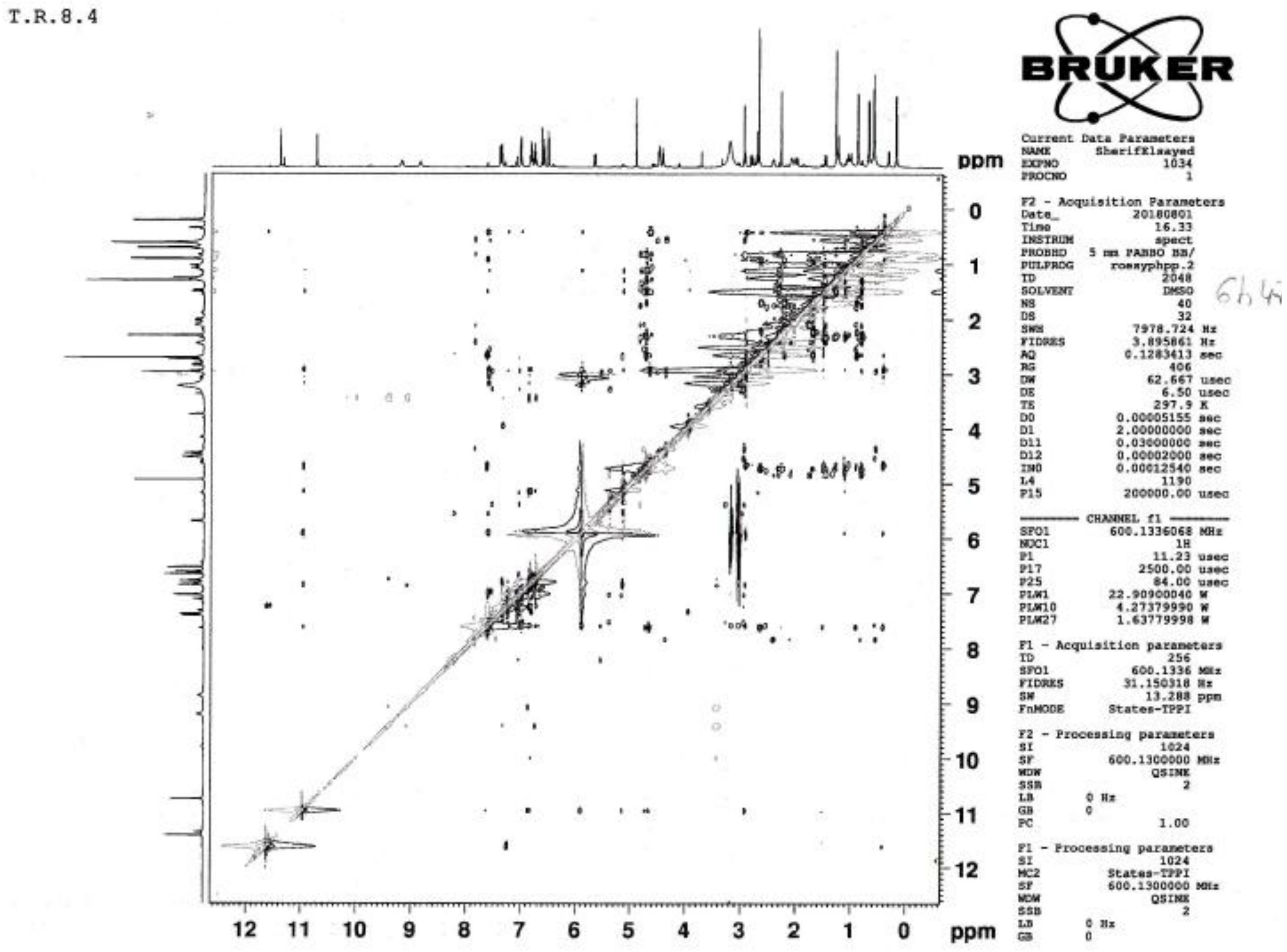

Figure S8. ROESY spectrum of **2** in DMSO-d_6

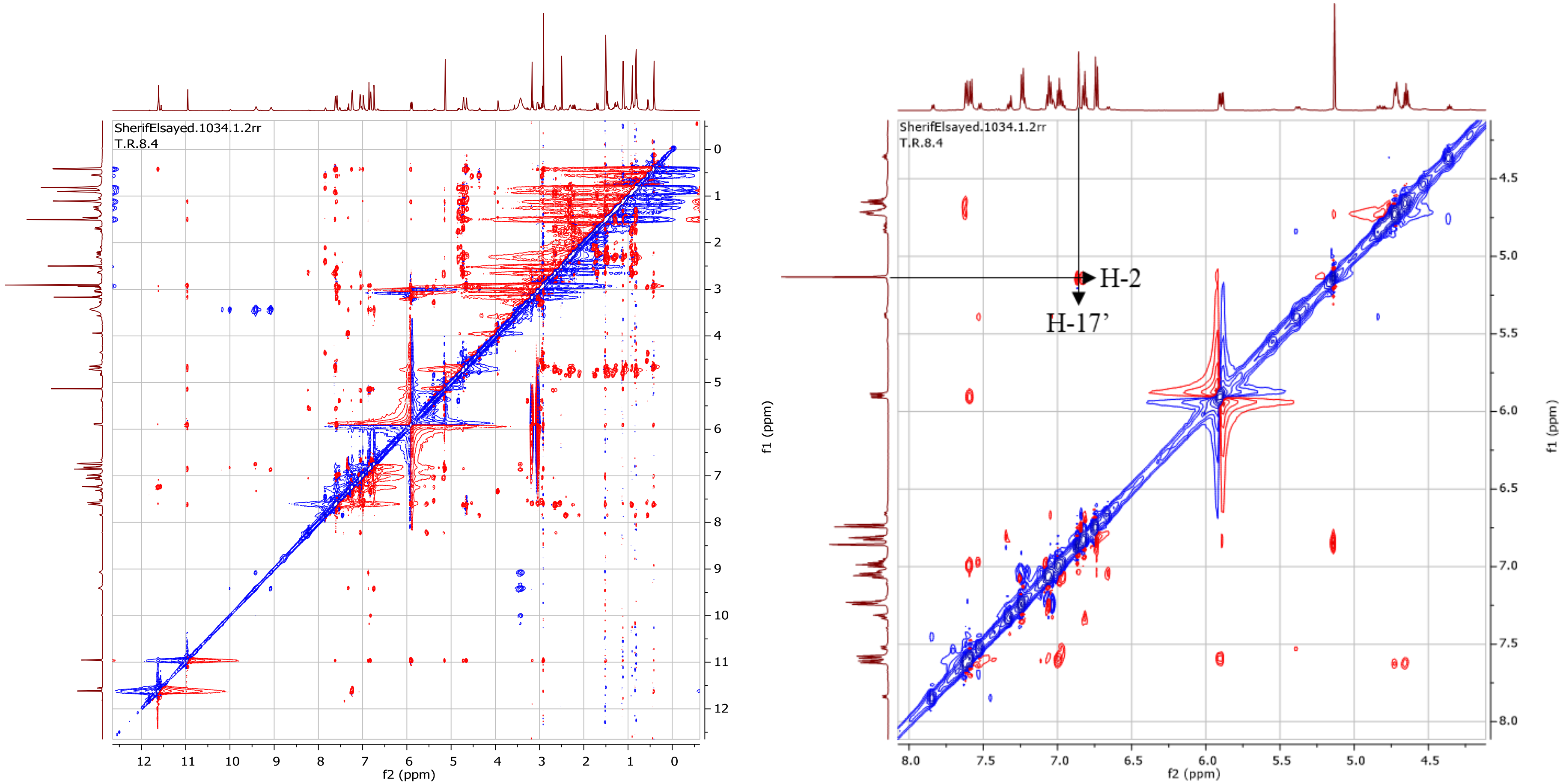

Figure S8a. ROESY spectrum of **2** in DMSO-d_6

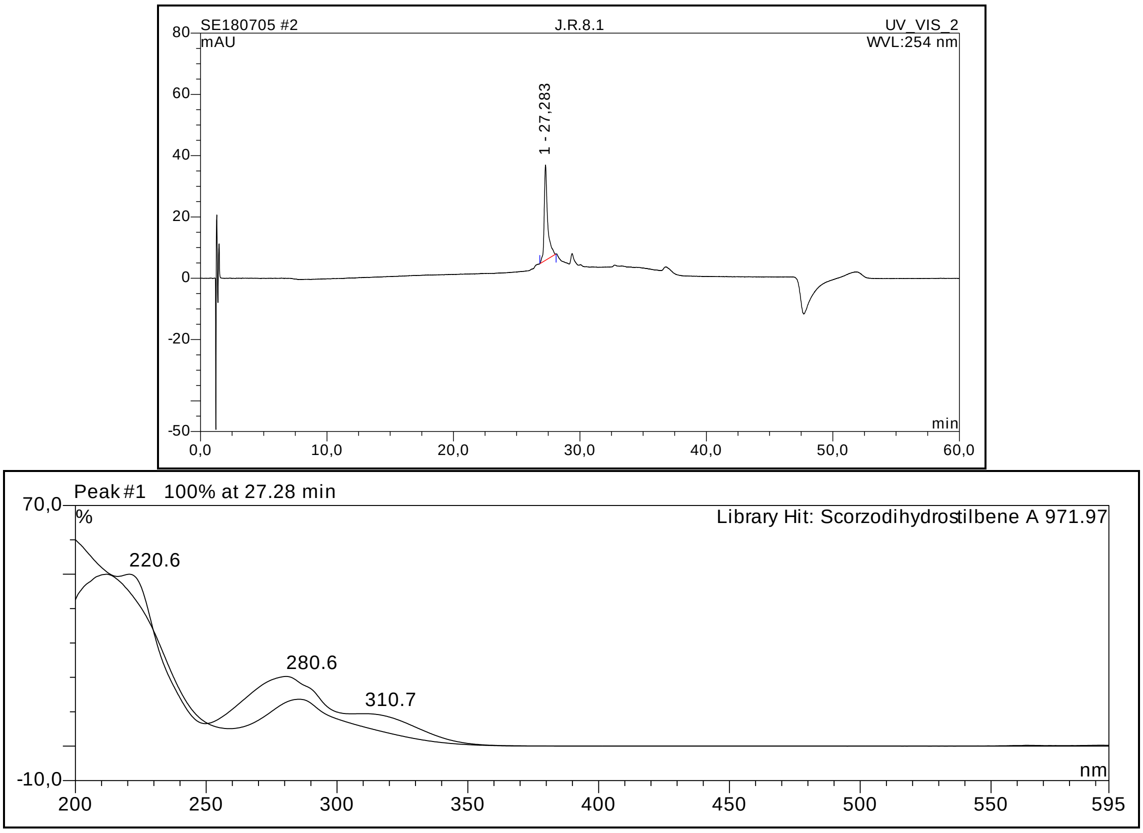

Figure S9. HPLC chromatogram of **4**

Mass Spectrum SmartFormula Report

Analysis Info

Analysis Name	D:\Data\Spektren 2018\PRO18HR000076.d	Acquisition Date	7/6/2018 10:04:23 AM
Method	tune_low_new.m	Operator	Peter Tommes
Sample Name	Sh. Elsayed J.R.8.1 (CH3OH)	Instrument	maXis 288882.20213
Comment	10 ul in 1 ml		

Acquisition Parameter

Source Type	ESI	Ion Polarity	Positive	Set Nebulizer	0.3 Bar
Focus	Not active	Set Capillary	4000 V	Set Dry Heater	180 °C
Scan Begin	50 m/z	Set End Plate Offset	-500 V	Set Dry Gas	4.0 l/min
Scan End	1500 m/z	Set Collision Cell RF	600.0 Vpp	Set Divert Valve	Source

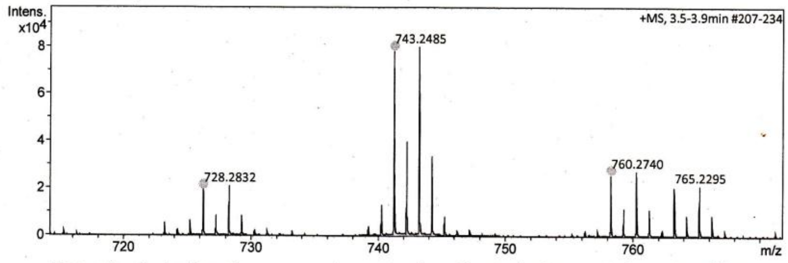

Meas. m/z	#	Ion Formula	m/z	err [ppm]	mSigma	# mSigma	Score	rdb	e⁻ Conf	N-Rule
726.2852	1	C36H49BrN5O6	726.2861	1.2	13.0	1	100.00	14.5	even	ok
	2	C33H41BrN15	726.2847	-0.7	14.2	2	99.89	20.5	even	ok
	3	C35H53BrNO10	726.2847	-0.6	43.6	3	59.33	9.5	even	ok
	4	C49H36N5O2	726.2864	1.6	470.2	4	0.00	34.5	even	ok
	5	C48H40NO6	726.2850	-0.3	470.3	5	0.00	29.5	even	ok
	6	C33H36N13O7	726.2855	0.4	493.4	6	0.00	22.5	even	ok
	7	C32H40N9O11	726.2842	-1.4	493.8	7	0.00	17.5	even	ok
741.2496	1	C50H29N8	741.2510	1.9	464.7	1	62.56	40.5	even	ok
	2	C49H33N4O4	741.2496	0.1	465.9	2	100.00	35.5	even	ok
	3	C48H37O8	741.2483	-1.7	467.2	3	41.98	30.5	even	ok
758.2749	1	C33H41BrN15O2	758.2746	-0.4	20.4	1	100.00	20.5	even	ok
	2	C36H49BrN5O8	758.2759	1.4	20.7	2	83.22	14.5	even	ok
	3	C35H53BrNO12	758.2746	-0.4	31.7	3	92.87	9.5	even	ok
	4	C48H45BrN3O	758.2741	-1.1	43.5	4	52.62	27.5	even	ok
	5	C49H36N5O4	758.2762	1.7	467.7	5	0.00	34.5	even	ok
	6	C48H40NO8	758.2748	-0.0	468.1	6	0.00	29.5	even	ok
	7	C45H32N11O2	758.2735	-1.8	474.7	7	0.00	35.5	even	ok
	8	C33H36N13O9	758.2753	0.6	492.2	8	0.00	22.5	even	ok

Figure S10. HRESIMS spectrum of **4**

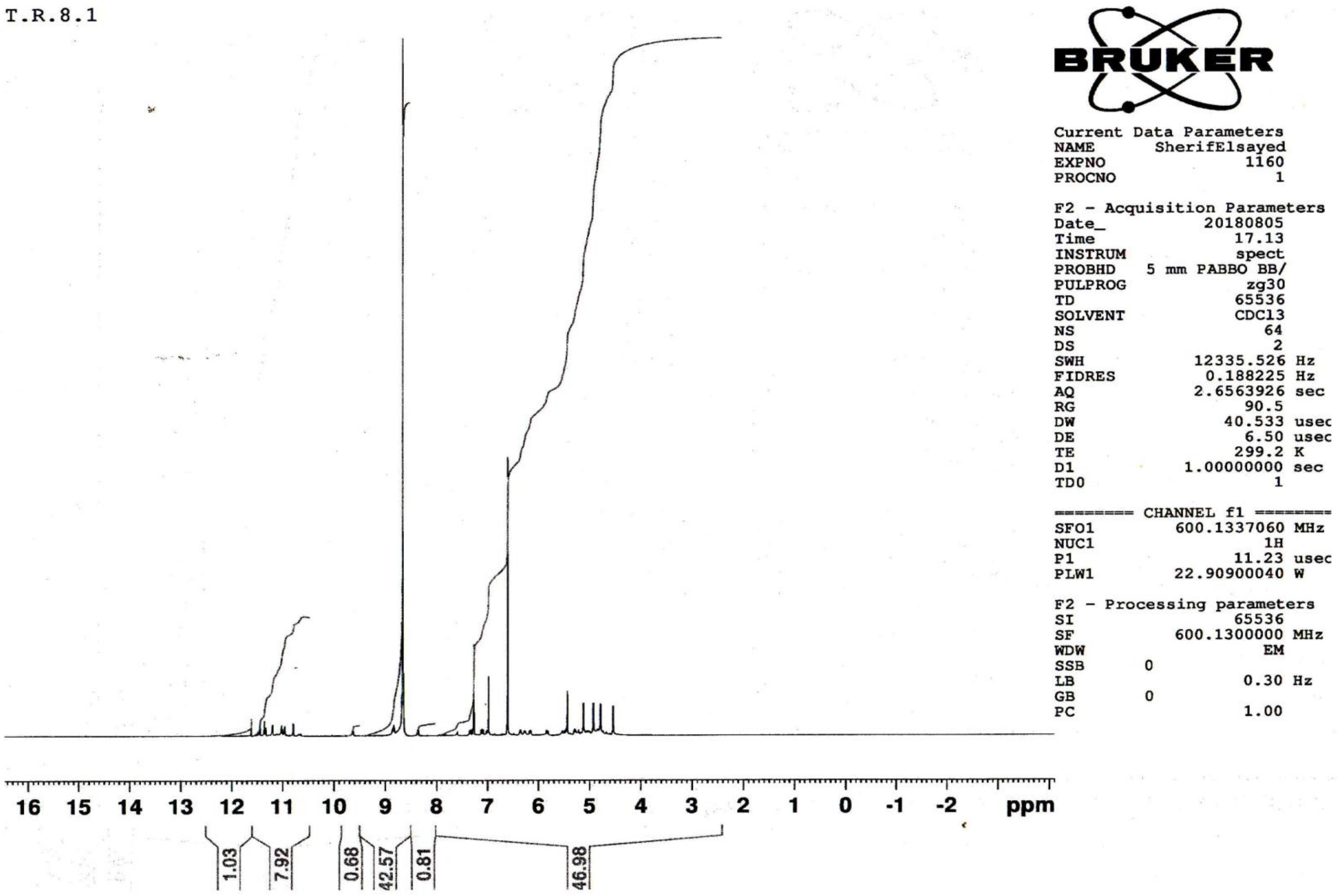

Figure S11. ^{1}H-NMR spectrum of **4** in $CDCl_3$

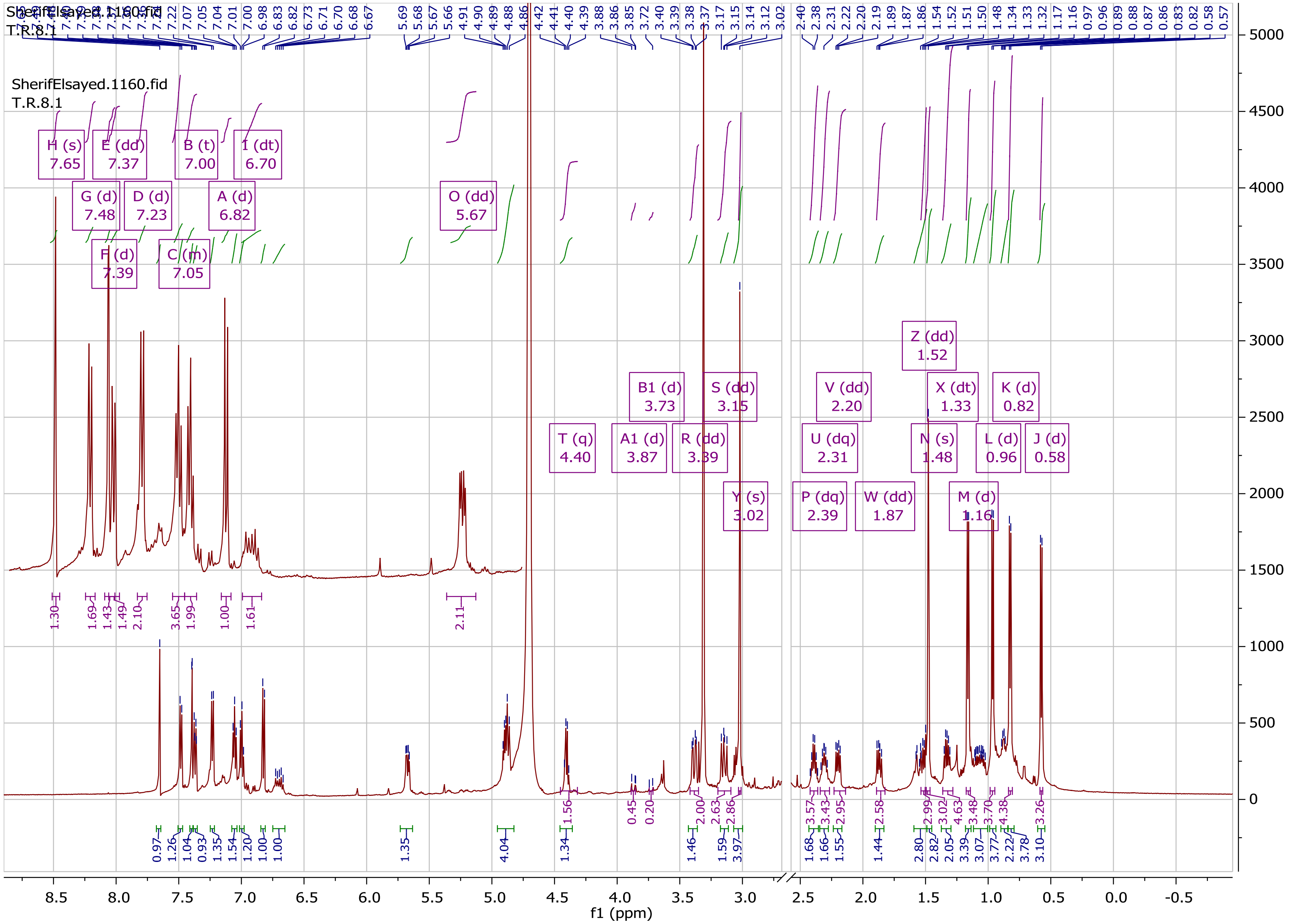

Figure S11a. ^{1}H-NMR spectrum of **4** in $CDCl_3$

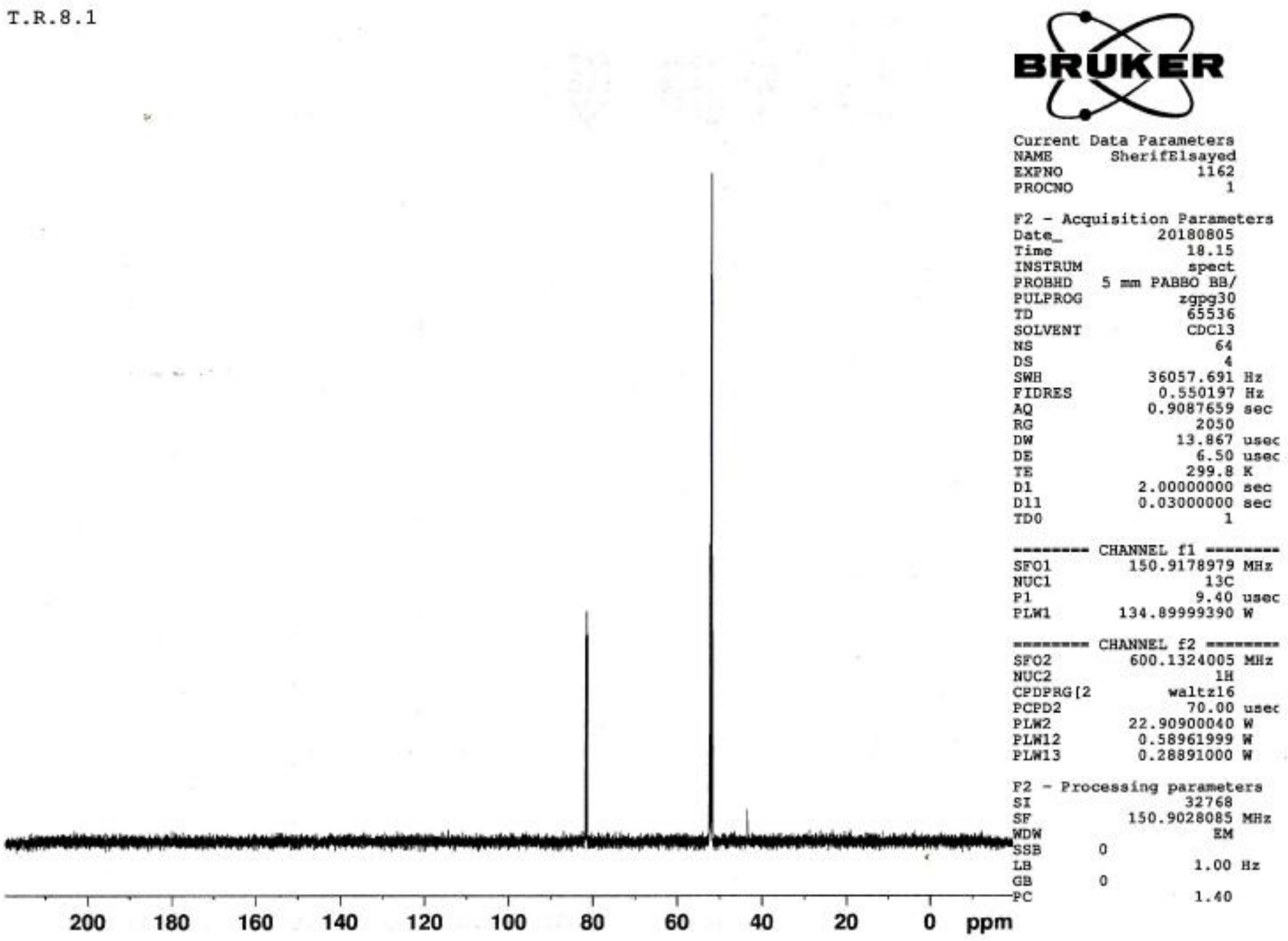

Figure S12. ^{13}C-NMR spectrum of **4** in $CDCl_3$

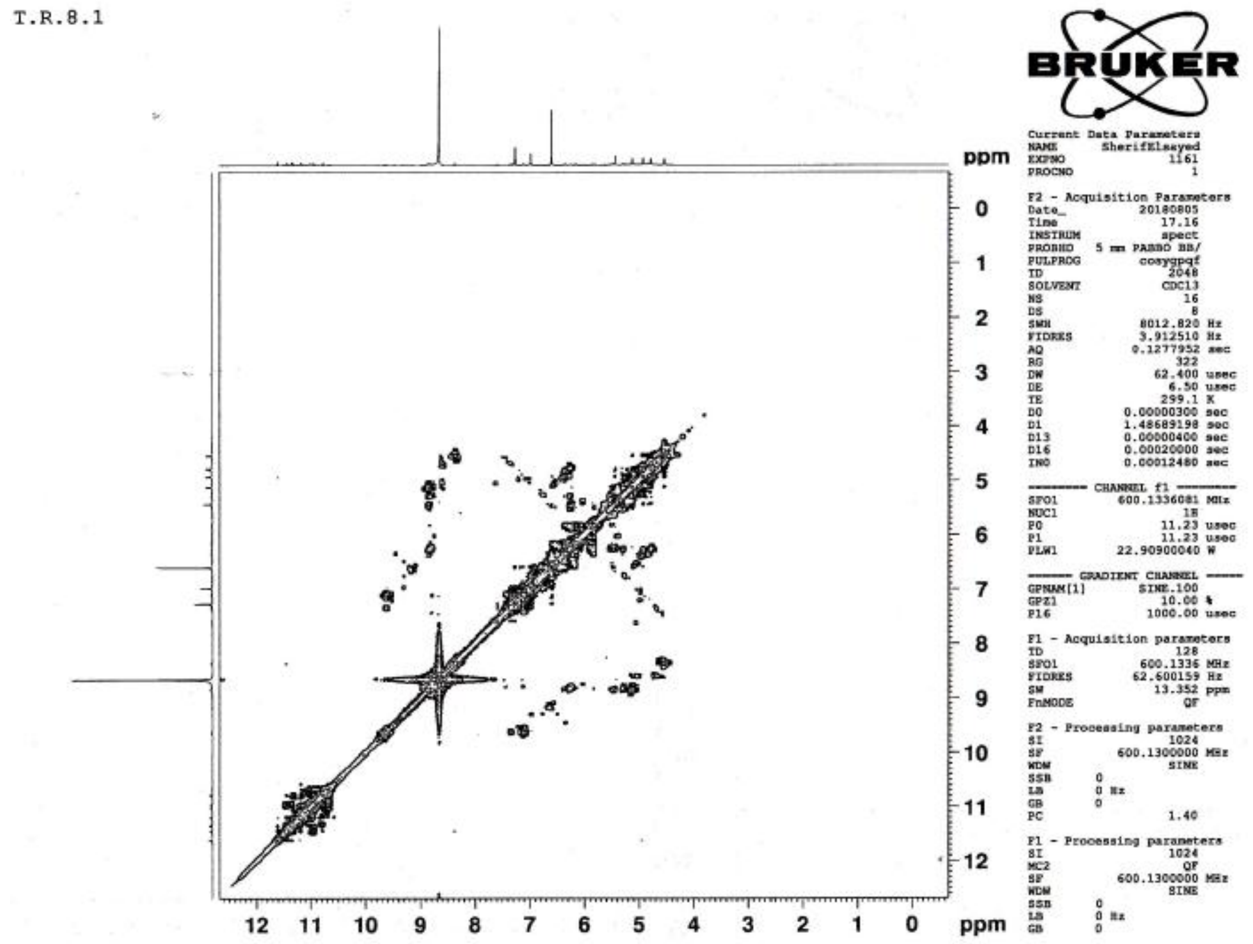

Figure S13. ^{1}H–^{1}H COSY spectrum of **4** in $CDCl_3$

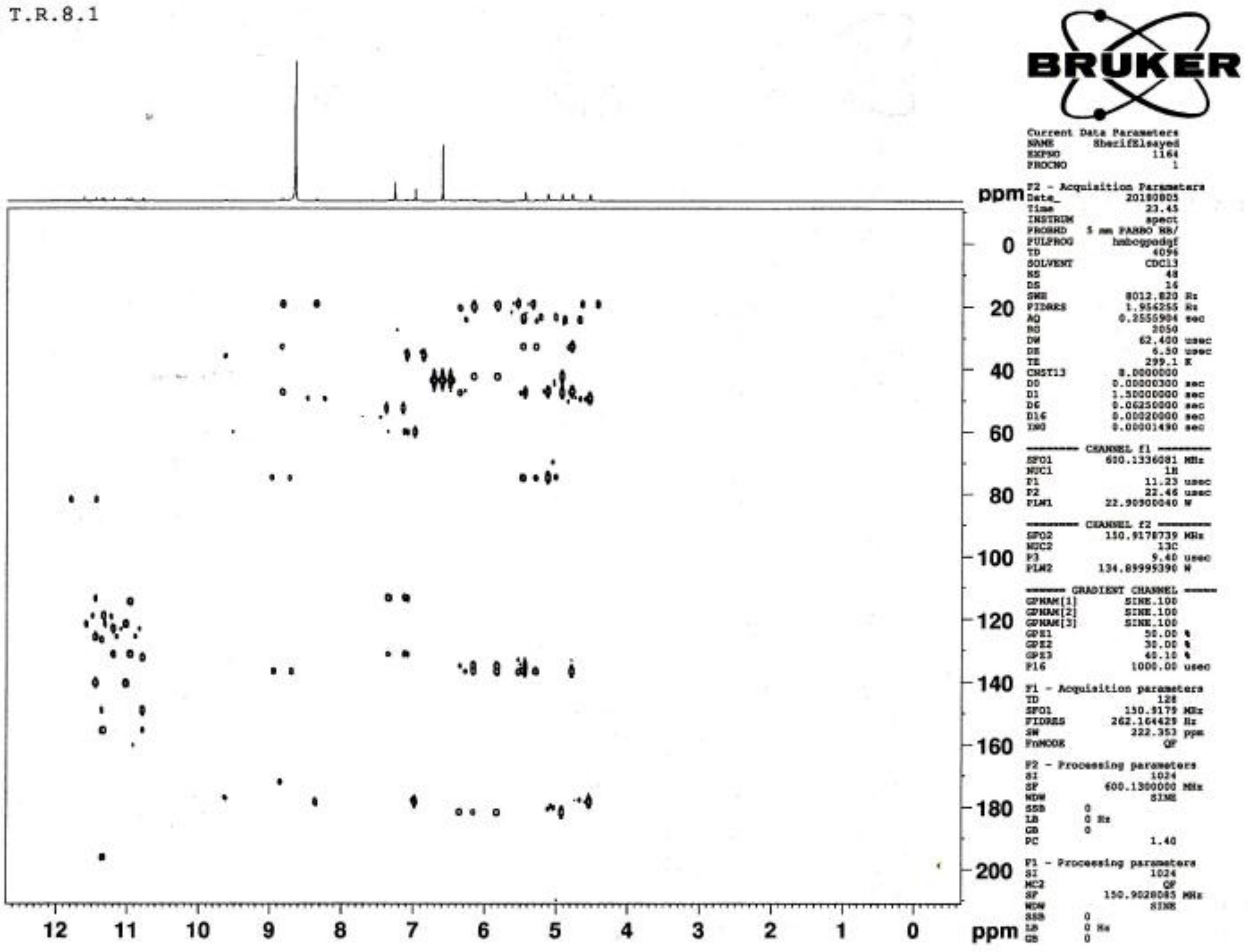

Figure S14. gHMBC spectrum of **4** in $CDCl_3$

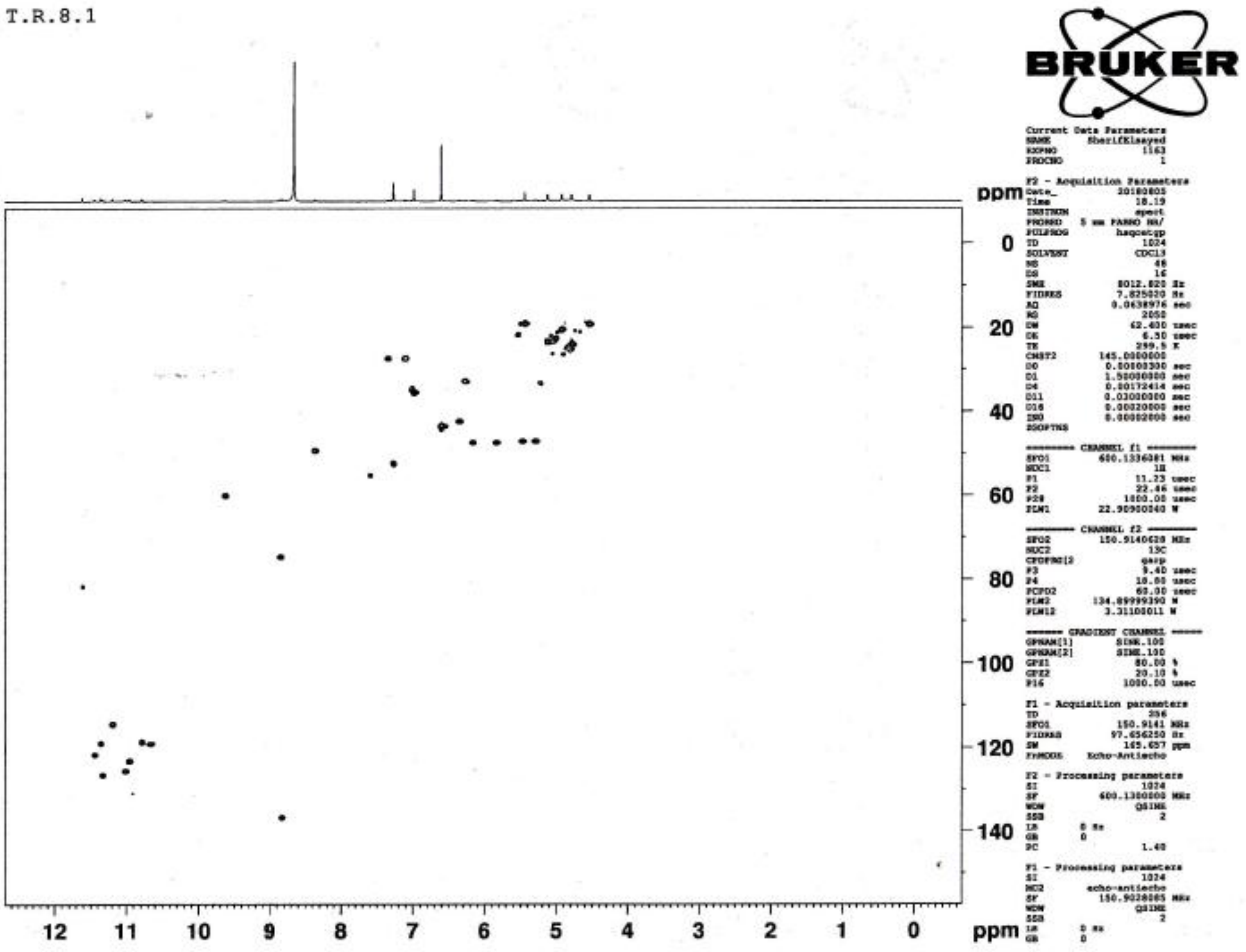

Figure S15. gHMQC spectrum of **4** in $CDCl_3$
